# PUM1-TRAF3 fusion protein activates non-canonical NF-κB signaling via rescued NIK in biliary tract cancer

**DOI:** 10.1038/s41698-024-00654-2

**Published:** 2024-08-01

**Authors:** Dawoon E. Jung, Mi-Kyoung Seo, Jung Hyun Jo, Kahee Kim, Chanyang Kim, Hyundeok Kang, Soo Been Park, Hee Seung Lee, Sangwoo Kim, Si Young Song

**Affiliations:** 1 Division of Gastroenterology, Department of Internal Medicine, Yonsei University College of Medicine, Seoul, Korea; 2 Institute of Gastroenterology, Yonsei University College of Medicine, Seoul, Korea; 3 Brain Korea 21 PLUS Project for Medical Science, Yonsei University College of Medicine, Seoul, Korea; 4 Department of Biomedical Systems Informatics, Yonsei University College of Medicine, Seoul, South Korea

**Keywords:** Bile duct cancer, Prognostic markers

## Abstract

Discovery and verification of diagnostic or therapeutic biomarkers for biliary tract cancer (BTC) is challenging owing to the low prevalence of the disease. Here, we identified and investigated the clinical impact of a fusion gene, Pumilio1-tumor necrosis factor receptor-associated factor 3 (PUM1-TRAF3), caused by 1;14 chromosomal translocation in BTC. PUM1-TRAF3 was initially identified in the RNA-sequencing of five BTC surgical tissues and confirmed by fluorescence in situ hybridization. Expression of the fusion gene was validated in an expanded cohort (5/55, 9.1%). Establishment and molecular assessment of PUM1-TRAF3 expressing BTC cells revealed that PUM1-TRAF3 activates non-canonical NF-κB signaling via NF-κB-inducing kinase (NIK). Abnormal TRAF3 activity, driven by competitive binding of PUM1-TRAF3 and TRAF3 to NIK, led to NIK rescue followed by P52/RelB nuclear translocation, all of which were reverted by an NIK inhibitor. The elevated expression of NIK and activated NF-κB signaling was observed in the PUM1-TRAF3-expressing regions of patient tissues. Expression of the PUM1-TRAF3 fusion was significantly correlated with strong NIK expression, which is associated with a poorer prognosis for patients with BTC. Overall, our study identifies a new fusion gene, PUM1-TRAF3, that activates NIK and non-canonical NF-κB signaling, which may be beneficial for developing precise treatment strategies for BTC.

## Introduction

Biliary tract cancer (BTC) encompasses a group of malignancies, including intrahepatic/extrahepatic cholangiocarcinoma (CCA) and gallbladder cancer^1^. Although relatively rare, BTC geographically varies in incidence rates are higher in Asian countries, including South Korea (6.3 cases per 100,000 people), than in western countries (<2 cases per 100,000 people)^2^. BTC is highly fatal, with a 5-year survival rate of <20%^3^. Although surgical resection is the only curative treatment, most cases of BTC are discovered at an advanced stage, and efficient early detection methods have not yet been developed. Even after curative resection, the recurrence rates are high (>30%)^4^. The treatment of choice for inoperable BTCs is a chemotherapy regimen comprising gemcitabine and cisplatin^5^; however, a standard for second-line chemotherapy has not been established. Meanwhile, research has shown that fibroblast growth factor receptor (FGFR) or neurotrophic tyrosine kinase receptor (NTRK) fusions and isocitrate dehydrogenase (IDH)-1/2 or BRAF (B-Raf Proto-Oncogene, Serine/Threonine kinase) mutations may be potential therapeutic targets in BTC^6–8^. However, FGFR and IDH inhibitors have only proven to be effective in approximately 20% of intrahepatic CCA patients with FGFR fusion and IDH mutations. Other druggable targets have proven difficult to identify^6,9,10^.

Recently, a few genomic studies have attempted to identify molecular markers to characterize BTC; these include Mucin17 (MUC17) deletions^7^, ARAF (A-Raf proto-oncogene, serine/threonine kinase) mutations^11^, and germline predisposing mutations^7,11,12^. However, more complex genomic events (e.g., alternative splicing and gene fusion) that can only be identified via transcript-level analysis have rarely been explored due to difficulties in acquiring fresh tumor samples for whole-transcriptome-level analysis^7,12^. Accordingly, we deemed that there may be an opportunity for the discovery of novel genomic markers in BTC by screening for such events via targeted RNA sequencing (RNA-seq) and polymerase chain reaction (PCR).

In this study, we conducted RNA sequencing of tissue samples from Korean BTC patients to identify BTC molecular markers and reported a fusion of the pumilio RNA-binding family member 1 (PUM1) and tumor necrosis factor (TNF) receptor-associated factor3 (TRAF3) genes. By establishing fusion protein-expressing cell lines, followed by functional assays, we confirmed the tumorigenic impact of the fusion gene and affected signaling pathways. Finally, we investigated the clinical value of the fusion gene as a therapeutic option.

## Results

### Discovery of gene fusion events

**Fig. 1:**
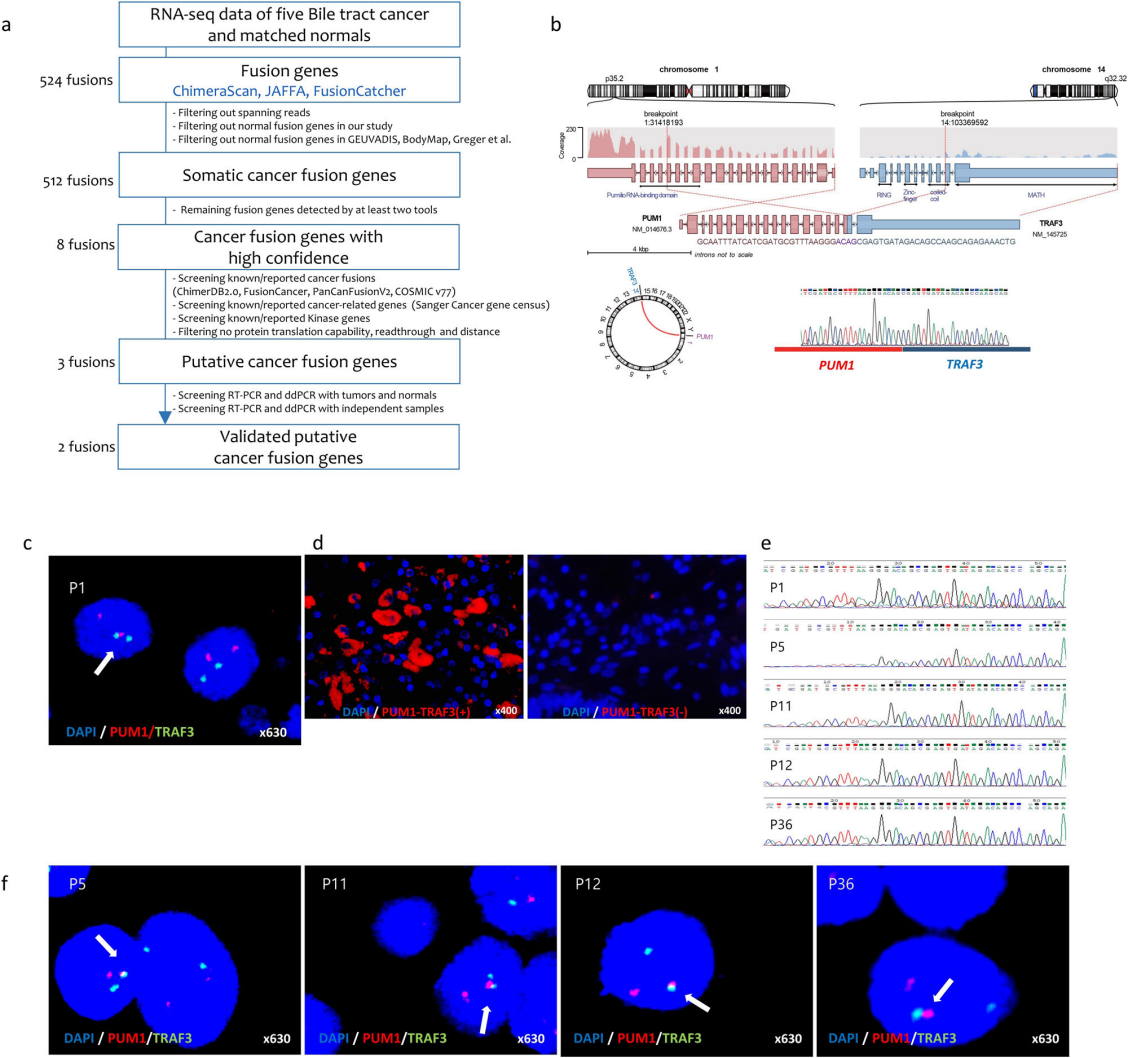
Discovery of the fusion gene - *PUM1-TRAF3.* **a** Workflow for fusion analysis in biliary tract cancer(BTC). **b** Schematic representation of *PUM1-TRAF3* fusion, involving *PUM1* (PUF domain, chromosome 1) and *TRAF3* (MATH domain, not the RING and Zinc finger domains; chromosome 14). Schematic diagrams and circos plots were visualized using Arriba. In the index patient (patient 1 (B01 or P1)), the chimeric junction between *PUM1* and *TRAF3* for *PUM1-TRAF3* fusion was confirmed via Sanger sequencing. **c** Fluorescent in situ hybridization (FISH) with two probes for *PUM1* (red) and *TRAF3* (green) in the P1 tissue slide. Arrow, fused signal. **d** In situ proximal ligation assay (PLA) was performed to visualize PUM1-TRAF3 protein expression in the P1 tissue slides using PUM1 rabbit polyclonal antibody and TRAF3 mouse monoclonal antibody; spot-like PLA signals were observed in the sample from the patient (B01 or P1) expressing the fusion protein (PUM1-TRAF3, red; DAPI, blue). **e** ddPCR product (P1, P5, P11, P12, and P36) was confirmed via Sanger sequencing. **f** FISH was performed with *PUM1* (red) and *TRAF3* (green) probes in the P5, P11, P12, and P36 tissue slides. Arrow, fused signal.

For initial discovery, we performed RNA sequencing of tissue samples from five Korean BTC patients; five matched normal bile duct tissues were also included (B01-B05, Supplementary Table S1). A customized bioinformatic pipeline was used for initial calling and strict filtration of gene fusions (Fig. 1a). Two high confidence gene fusions, PUM1-TRAF3 and ASH1L-DOCK7, which passed the filtration steps were noted in one patient (B01 or P1) with intrahepatic CCA. PUM1-TRAF3 was in-frame whereas ASH1L-DOCK7 was out-frame; thus, we further analyzed and validated with PUM1-TRAF3 (Fig. 1b, Supplementary Fig. 1). Neither of the two fusion events had been reported for any type of cancer. Next, we analyzed publically available RNA sequencing datasets for BTC^7^ (*n* = 7). To determine whether the fusion event is recurrent in other cancers, we sourced further data from The Cancer Genome Atlas (TCGA) for cholangiocarcinoma (CHOL; *n* = 36) and cancers of nearby bile duct tissues, including hepatocellular carcinoma (LIHC; *n* = 374) and pancreatic ductal adenocarcinoma (PDAC; *n* = 183). We did not find any evidence of PUM1-TRAF3 expression.

To validate the PUM1-TRAF3 fusion, we conducted PCR using primers designed to cover putative breakpoints (Supplementary Table S2: PT1 forward 5′-TGT ATG GCT GCC GTG TTA TC-3′ and reverse 5′-ATG TCG TGC ACA CTC AGC AT-3′). Subsequent Sanger sequencing successfully confirmed the fusion in patient P1. PUM-TRAF3 is an in-frame transcript and comprises 2988 bp of PUM1 (out of 3558 bp located at chr1:p35.2) and 744 bp of TRAF3 (out of 1704 bp at chr14:q32.32) (Fig. 1b, Supplementary Fig. 2). In the PUM1-TRAF3 transcript, pumilio RNA-binding domain of PUM1 partially remained, whereas the MATH domain of TRAF3, which is required for physical binding to NF-κB-inducing kinase (NIK), was preserved. The other two domains of TRAF3 (i.e., the RING finger and zinc finger domains), which are required for ubiquitination and degradation of NIK, were lost.

We further validated the fusion events at the genomic and proteomic levels. The chromosomal translocation t(1;14)(p35.2;q32.32) was confirmed by fluorescence in situ hybridization (FISH) assay using PUM1 and TRAF3 probes (Fig. 1c). To verify the presence of endogenous PUM1-TRAF3 fusion protein in patient tissue, we performed an in situ proximal ligation assay (PLA) to detect and visualize the protein complexes. Using the PUM1 rabbit polyclonal antibody and TRAF3 mouse monoclonal antibody (Supplementary Table S3), we visually observed the fusion protein as a spot-like pattern of PLA signals in patient tissue slides via fluorescence microscopy (Fig. 1d). These results confirm that the PUM1-TRAF3 events occur via genomic translocation and are actively expressed to generate the fusion protein in BTC.

### Frequency of PUM1-TRAF3 fusion in BTC

We screened additional BTC cases to determine the frequency of PUM1-TRAF3 expression. We conducted droplet digital PCR (ddPCR) analyses of 26 frozen tissue pairs (normal and cancer tissue), including the 3 RNA-seq tissue pairs (B01 or P1, B02 or P3, B03 or P4), and 29 formalin-fixed paraffin-embedded (FFPE) samples. We detected signals in 28 samples (considered positive when the concentration (copies/µl) was 1 or above (Supplementary Fig. 3)); no fusion transcript was detected in the paired-normal tissues from 26 patients (P1-P26). To eliminate false-positive signals, PCR products were analyzed via Sanger sequencing (Fig. 1e) and FISH was performed with PUM1 and TRAF3 probes (Fig. 1f). PUM1-TRAF3 fusion and its expression in five samples were confirmed both by Sanger sequencing and FISH, which indicated an overall frequency of 9.1% (5 out of 55). There were no significant differences in the baseline characteristics of the fusion-positive group (*n* = 5) and the fusion-negative group (*n* = 50); this may be due to the small number of patients in the fusion-positive group (Supplementary Tables S4 and S5).

### PUM1-TRAF3 expression elevates NIK expression and activates non-canonical NF-κB pathway

We analyzed gene and protein expression via PCR and western blotting, respectively, to examine the expression of PUM1-TRAF3 in BTC cell lines. We utilized nine BTC cell lines, namely SNU245, SNU308, SNU478, SNU869, SNU1079, SNU1196, Hucct-1, OZ, and KKU-100. We found that PUM1-TRAF3 mRNA and protein expression was detected only in Hucct-1 cells (Supplementary Fig. 4).

**Fig. 2:**
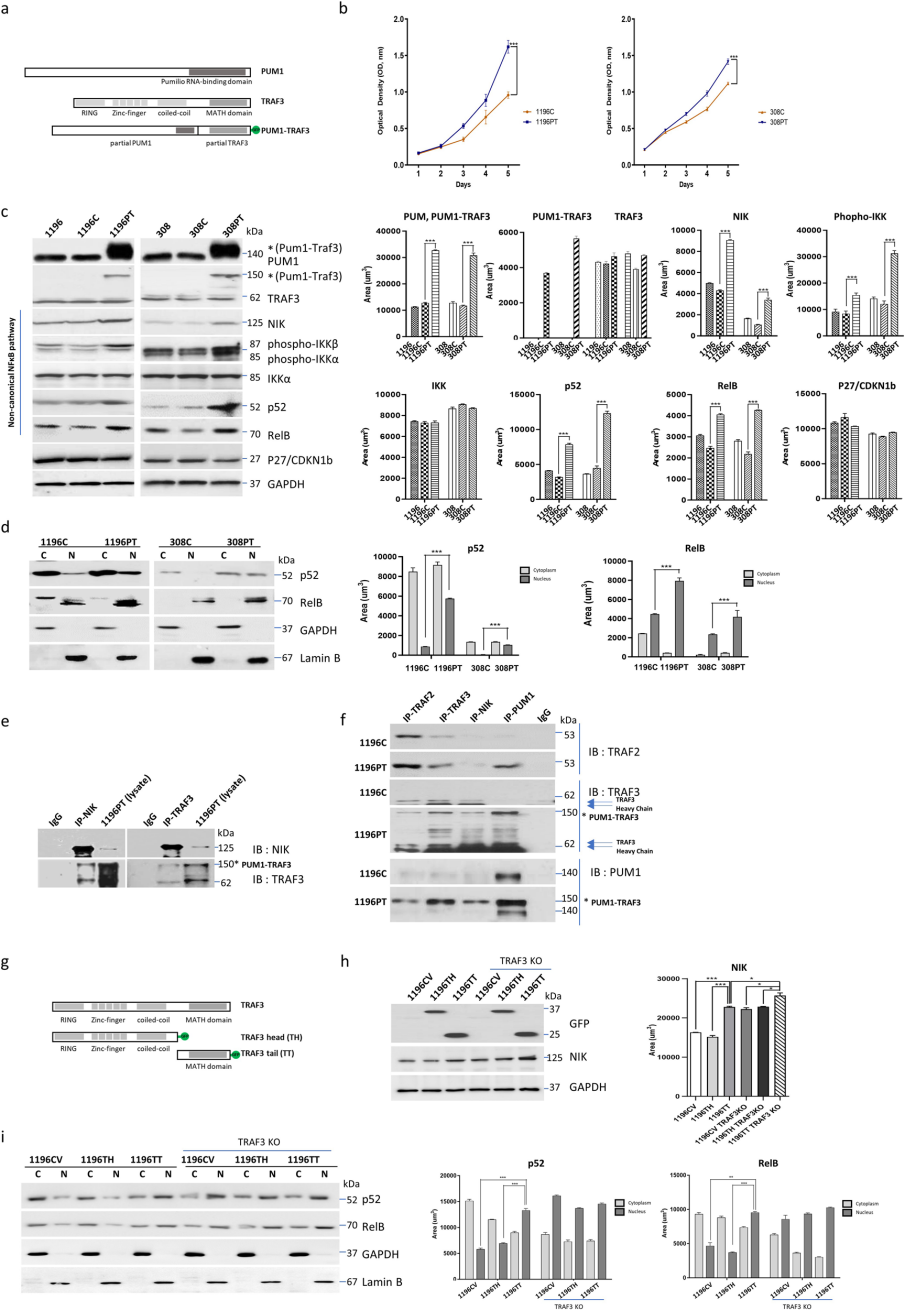
*PUM1-TRAF3* expression elevates NIK expression and activates non-canonical NF-κB pathway. **a** Diagram of full-length *PUM1, TRAF3*, and *PUM1-TRAF3*(PT) fusion constructs. **b** *PUM1-TRAF3* expression enhanced cell proliferation by 69.06 ± 1.90% (*p* < 0.001) in SNU1196 cells and by 26.88 ± 0.77% (***p < 0.001) in SNU308 cells. Data presented as mean ± S.D. **c** *PUM1-TRAF3* expression elevates NIK and non-canonical NF-κB pathway associated proteins. The protein expression was assessed by area (***p < 0.001). Data presented as mean ± S.D. **d** *PUM1-TRAF3*-induced NF-κB activation results in the translocation of NF-κB2(p52) and RelB (C, cytoplasmic fraction; N, nucleic fraction). The protein expression was assessed by area (***p < 0.001). Data presented as mean ± S.D. **e** PUM1-TRAF3 interacts NIK: Protein-protein interactions among NIK, TRAF3, and truncated TRAF3 from PUM1-TRAF3 were assessed in 1196PT via immunoprecipitation. **f** PUM1-TRAF3 interacts TRAF2, TRAF3, and NIK: Protein-protein interactions among NIK, TRAF2, TRAF3, and PUM1 were assessed in 1196C and 1196PT via immunoprecipitation (IP, immunoprecipitation; IB, immunoblot). **g** Diagram of full-length *TRAF3*, and partial *TRAF3* constructs. **h** *TRAF3* tail (TT)-transfected cells elevates NIK expression. Analysis NIK expression of partial *TRAF3*-transfected cell proteins (CV, control vector; TH, *TRAF3* head; TT, *TRAF3* tail). The protein expression was assessed by area (***p < 0.001). Data presented as mean ± S.D. **i** *TRAF3* tail (TT)-transfected cells enhance the translocation of NF-κB2 (*p52*) and RelB (C, cytoplasmic fraction; N, nucleic fraction). The protein expression was assessed by area (**p < 0.01; ***p < 0.001). Data presented as mean ± S.D.

To assess the functional effects of the PUM1-TRAF3 fusion protein, we constructed a plasmid expressing PUM-TRAF3 tagged with green fluorescence protein (GFP) as descripted in the method section (Fig. 2a). We established PUM1-TRAF3-expressing cell lines (PT cell lines) by stably transducing PUM1-TRAF3 into the BTC cell lines, SNU308 (308PT) and SNU1196 (1196PT); both cell lines normally expressed both the PUM1 and TRAF3 genes. We found that expression of the PUM1-TRAF3 fusion protein led to a significant increase in the proliferation of 308PT and 1196PT cells (69.16 ± 1.90% and 26.88 ± 0.77%, respectively, *p* < 0.001) compared to that of cells treated with the control vector (C) cell lines of 308C and 1196C (Fig. 2b).

Next, we examined the expression and activity of the downstream components of PUM1-related and TRAF3-related signaling pathways, using western blotting, to determine the oncogenic roles of the involved genes (Fig. 2c, Supplementary Fig. 5). We found that the expression of p27/CDKN1b (a cell cycle inhibitor directly repressed by PUM1) was comparable between the PT and C cell lines, suggesting that the partial PUM1-driven signaling does not exert oncogenic effects (Fig. 2c). Meanwhile, the expression of NIK and its downstream non-canonical nuclear factor kappa-light-chain-enhancer of activated B cells (NF-κB) pathway, including phospho-IKKα (activated IKKα), Nuclear Factor Kappa B Subunit 2 (NF-κB2 or p52), and RelB proto-oncogene, NF-κB Subunit (RelB), was markedly elevated in PT cells (Fig. 2c). It is well known that NIK activates the non-canonical NF-κB pathway by processing the precursor NF-κB2 (p100) to the mature form p52, and that this activation induces the formation of RelB/p52 heterodimer, which translocate to the nucleus^13^. We observed that PT expression led to nuclear translocation and elevation of nuclear p52 and RelB (Fig. 2d). The NF-κB pathway is known to induce epithelial-mesenchymal transition (EMT)^13^ and PT expression induced EMT associated proteins including TWIST and Snail/Slug (Supplementary Fig. 5a). We also observed significant increase in cell motility both in the migration (67.28 ± 12.58% and 69.71 ± 17.79%, *p* < 0.001) and invasion (150.91 ± 6.56% and 75.38 ± 4.58%, *p* < 0.001) of PT cells (Supplementary Fig. 5b). In soft agar colony formation assay, we noted marked increase in the sizes of colonies of the PT cell lines, while the number of colonies remained similar (Supplementary Fig. 5b).

Since NIK controls the non-canonical NF-κB pathway via the negative regulator TRAF3^14,15^, we hypothesized that truncated TRAF3 undergirds the major oncogenic functions of the fusion protein^16–19^. We investigated whether the observed increase in NIK expression was related to the known regulatory mechanism of TRAF3, which forms a complex with TNF Receptor-Associated Factor 2 (TRAF2) and induces NIK degradation. The immunoprecipitation analysis showed that both the naïve and truncated forms of TRAF3 from PT bound to NIK and TRAF2 (Fig. 2e, f). To examine whether the truncated forms of TRAF3 interfere with the function of naïve TRAF3, we generated TRAF3 knockout (KO) cells using CRISPR/Cas9 and transfected them with TRAF3 head (TH) and TRAF3 tail (TT) transcripts (Fig. 2g). Cells transfected with TRAF3 tail (1196TT) enhanced NIK expression and further translocated p52 and RelB comparable to cells lacking TRAF3 expression (Fig. 2h, i). Thus, we observed that the partial TRAF3 from PUM1-TRAF3 binds to NIK and TRAF2 but fails to degrade NIK due to the lack of head parts of TRAF3, leading to the rescue of NIK from degradation that resulted in NIK increment.

### PUM1-TRAF3 induced NIK increase drives the constitutive activation of non-canonical NF-κB signaling

To examine whether PT expression interferes with naïve TRAF3 activity and activates the non-canonical NF-κB pathway, we generated TRAF3 knockout (KO) cells expressing control vector or PUM-TRAF3.

**Fig. 3:**
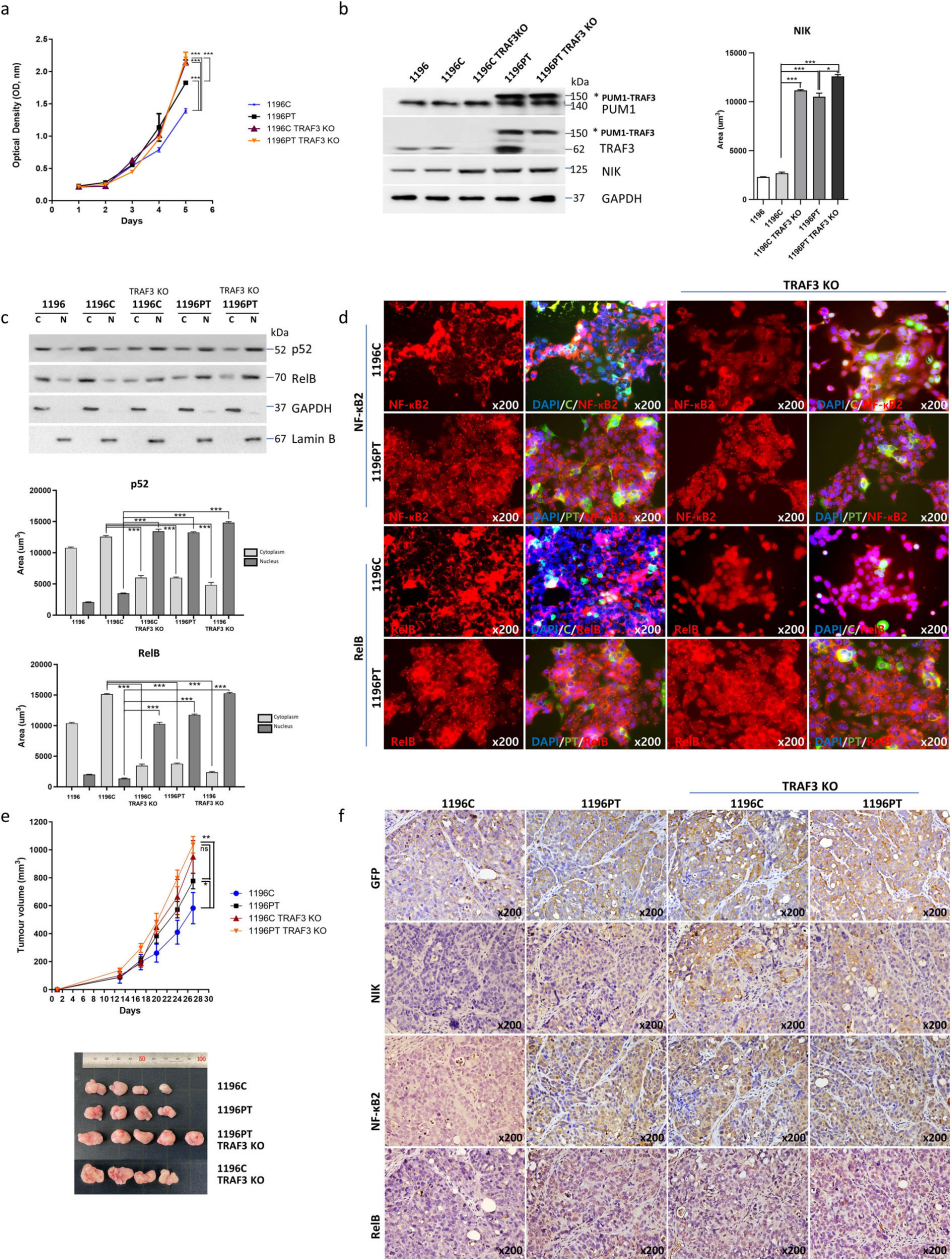
*PUM1-TRAF3* expression activates the non-canonical NF-κB pathway and induces NF-κB2 and RelB translocation. **a** Both 1196C and 1196PT cells with silenced *TRAF3* gene expression (*TRAF3* KO) showed increased proliferation (63.55 ± 3.65% in 1196C *TRAF3* KO, 37.36 ± 1.22% to 68.72 ± 8.55% in 1196PT and 1196PT *TRAF3* KO, respectively (***p < 0.001). Data presented as mean ± S.D. **b** Proteins differentially expressed in *PUM1-TRAF3*-transduced cells were analyzed; these included PUM1, TRAF3, PUM1-TRAF3, and NIK (***p < 0.001). The protein expression was assessed by area (***p < 0.001). Data presented as mean ± S.D. **c** PUM1-TRAF3-induced NF-κB activation with or without naïve *TRAF3* results in the translocation of NF-κB2(*p52*) and RelB (C, cytoplasmic fraction; N, nucleic fraction). The protein expression was assessed by area (***p < 0.001). Data presented as mean ± S.D. **d** Immunofluorescence staining for NF-κB2 and RelB in SNU1196 control vector-transduced and SNU1196 *PUM1-TRAF3*-transduced cells. *TRAF3* knockout enhances NF-κB2 and RelB translocation in control vector and *PUM1-TRAF3*-transduced cells. (DAPI, blue; Green, PT or C; Red, NF-κB2 or RelB). **e** *PUM1-TRAF3*-transduced and control vector (C)-transduced cells (5 × 10^6^ cells/site) with or without *TRAF3* expression were injected subcutaneously into four groups of mice, and tumor size was measured every 7 days (volume = length × width^2^ × 1/2). At 27 days post injection, the average volumes of tumors were 582.6 ± 223.6 mm^3^, 802.8 ± 167.0 mm^3^, 898.9 ± 349.7 mm^3^ (*p* < 0.05), and 1019.9 ± 156.6 mm^3^ (*p* < 0.01) in 1196C, 1196PT, 1196C *TRAF3* KO, and 1196PT *TRAF3* KO mice, respectively (*P < 0.05; **P < 0.01; ns no significance). Data presented as mean ± S.D. **f** Immunohistochemical staining of GFP (control vector or *PUM1*-*TRAF3*), NIK, NF-κB2(p52) and RelB in mouse tumor tissues in C- and PT-expressing cell lines.

The silencing of TRAF3 gene expression enhanced proliferation in both 1196C and 1196PT cells compared to the TRAF3 expressing cells (63.55 ± 3.65% in 1196C TRAF3 KO, and 37.36 ± 1.22% to 68.72 ± 8.55% in 1196PT and 1196PT TRAF3 KO, respectively, *p* < 0.001) (Fig. 3a). We analyzed NIK protein and gene expression in the TRAF3-silenced PT and C cell lines via western blotting and RNA sequencing, respectively. The expression of NIK was elevated in C cells with silenced TRAF3 expression, whereas NIK was comparably expressed in PT cells regardless of TRAF3 expression (Fig. 3b, Supplementary Fig. 6a). Along with NIK expression, Octamer-Binding Transcription Factor (OCT4) expression was also elevated (Supplementary Fig. 6b, c); Vazquez-Santillan et al. previously reported that NIK expression enhances the expression of cancer stem cell-related genes, including OCT4, in breast cancer^20^. It has been suggested that tumors contain minor fractions of subpopulations, such as cancer stem cells, that can initiate and sustain tumor growth via self-renewal and even differentiate into different phenotypes^21^. We performed a sphere formation assay to validate whether PT can enhance the features of cancer stem cells and found that, along with TRAF3-silenced C cells, the sphere-forming ability of PT transduced cells was enhanced, regardless of TRAF3 expression (120.7 ± 22.0, 323.0 ± 44.9, 270.67 ± 43.5, and 287.7 ± 12.3 spheres in 1196 C, 1196 C TRAF3 KO, 1196PT, and 1196PT TRAF3 KO cells, respectively, *p* < 0.001) (Supplementary Fig. 6d). Next, we assessed the expression of a putative BTC cancer stem cell surface marker, CD133^+^^22^. Results indicated that CD133^+^ expression increased in the TRAF3-silenced C cells and PT cells (0.78%, 7.3%, 12.7%, and 12.0% in 1196C, 1196C TRAF3 KO, 1196PT, and 1196PT TRAF3 KO cells, respectively) (Supplementary Fig. 6e).

We examined the subcellular localization of p52 and RelB in the PT and C cell lines. Comparable to TRAF3 KO cells, and regardless of TRAF3 expression, 1196PT cells showed constitutive expression of activated NIK and induced nuclear translocation of p52 and RelB (Fig. 3c). Strong expression of both NF-κB2 and RelB in nucleus was also observed upon immunofluorescence staining of PT cells, regardless of TRAF3 expression (Fig. 3d, expanded panels in Supplementary Fig. 7). Moreover, PT and naïve TRAF3 worked competitively. In the absence of TRAF3, as observed in the TRAF3 KO condition, there was a notable absence of TRAF inhibitory effect on NIK, consequently leading to NIK activation (Fig. 3b). In contrast, when both PT and TRAF3 were present, there was competition between the two, potentially leading to a reduction in TRAF3-mediated inhibition of NIK. This competitive interaction could result in enhanced NIK activation and subsequent induction of the translocation of p52 and RelB compared to when 1196PT was expressed alone (Fig. 3c, d). Taken together, we propose that the PUM1-TRAF3 gene fusion drives the activation of non-canonical NF-κB signaling primarily via NIK activation, with the absence of TRAF3 dismissing its regulatory role and allowing for unimpeded action of NIK.

Next, we assessed tumorigenicity by subcutaneously injecting tumor cells from the TRAF3-expressing or KO cell lines with PT or C into mice (*n* = 5 per group. At 27 days post injection, tumor formation was observed in four out of five mice in all groups, except for 1196PT TRAF3 KO, and the average volumes of tumors were 582.6 ± 223.6 mm^3^, 766.9 ± 145.1 mm^3^, 998.9 ± 235.1 mm^3^, and 1101.3 ± 127.8 mm^3^ in 1196C, 1196PT, 1196C TRAF3 KO, and 1196PT TRAF3 KO mice, respectively (Fig. 3e). We examined the expression of GFP (control vector or PUM1-TRAF3), NIK, p52 and RelB in tumors using immunohistochemical analyses. We observed elevated expression NIK along with p52 and RelB (primarily in the nucleus) in mouse tumor tissues expressing the PUM1-TRAF3 fusion protein, regardless of TRAF3 expression (Fig. 3f). Taken together, these results suggested that PUM1-TRAF3 elicits the constitutive activation of non-canonical NF-κB signaling and increases cell proliferation via truncated TRAF3-mediated accumulation of NIK.

### Inhibition of NIK expression causes a potential reversal of PUM1-TRAF3-induced NF-κB activation

**Fig. 4:**
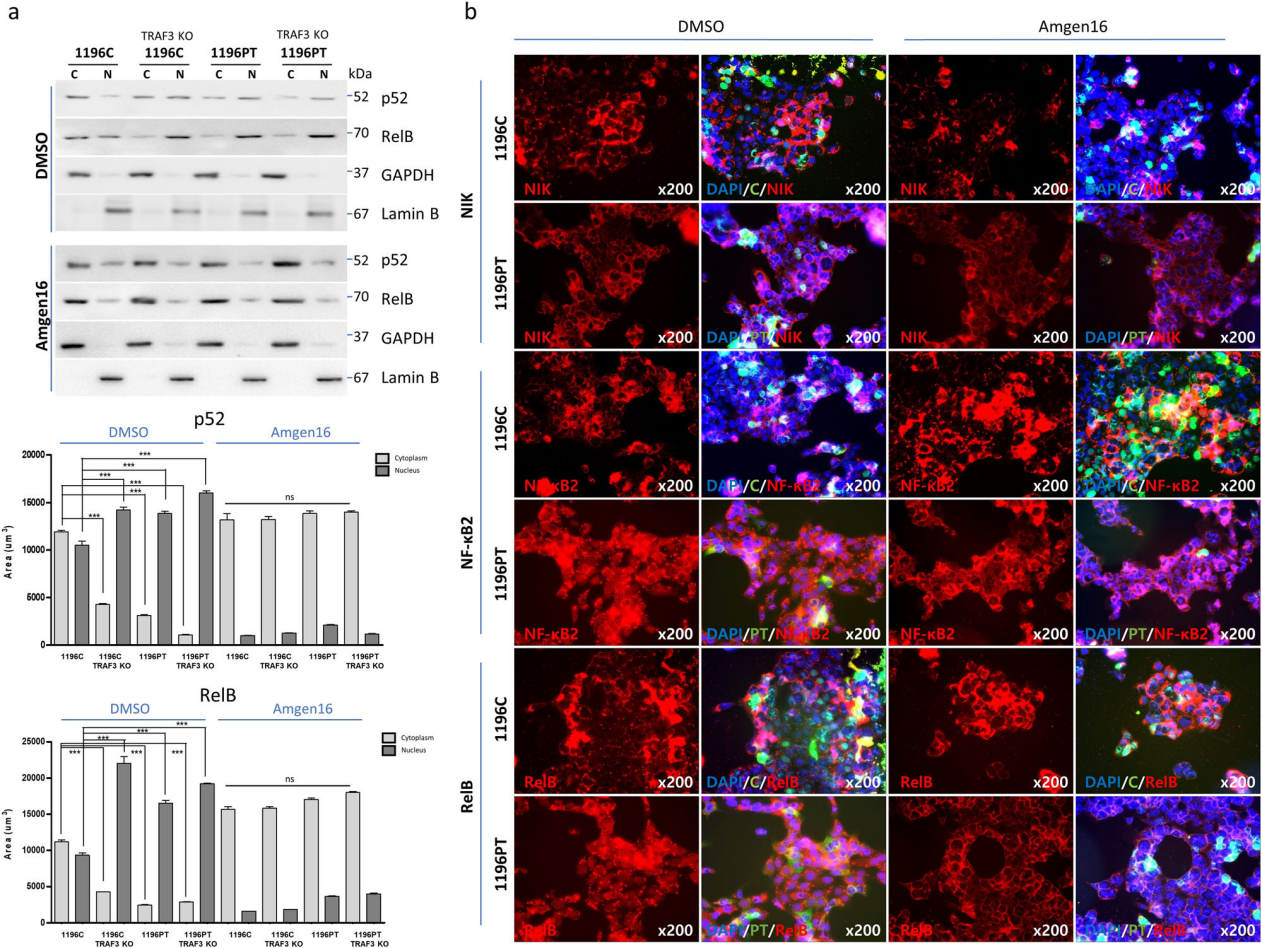
Inhibition of NIK expression reverted PUM1-TRAF3-induced activation of the non-canonical NF-κB pathway. **a** Cells treated with DMSO or NIK inhibitor, 10 µM Amgen16, and nuclear and cytoplasmic fractions were isolated via cell compartment fractionation, and p52 and RelB were assessed (C, cytoplasmic fraction; N, nucleic fraction; ***P < 0.001; ns no significance). Data presented as mean ± S.D. **b** NIK inhibition diminishes PUM1-TRAF3-induced activation. NIK, NF-κB2, and RelB were visualized in C-transduced cells, TRAF3 knockout cells, PUM1-TRAF3-transduced cells, and TRAF3 knockout PUM1-TRAF3-transduced cells treated with DMSO or 1 µM Amgen16, via immunofluorescence staining. (DAPI, blue; green, PT or C; red, NIK, NF-κB2 or RelB).

We investigated whether the fusion protein-induced NF-κB signaling pathway could be reverted by inhibition of NIK. Both the PT and C cell lines as well as the original cell line SNU1196 were treated with NIK inhibitors, B022 and Amgen 16, at five-fold serial dilutions, at concentrations ranging from 0 µM to 100 µM (Supplementary Fig. 8a). Treating cells with two doses (10 µM and 50 µM), 50 µM Amgen 16 or B022 sufficiently inhibited NIK expression (Supplementary Fig. 8). To minimize the cytotoxicity and off-target effects associated with NIK inhibitors, cells were treated with 10 µM Amgen 16 or B022 for western blotting. While NIK levels were not notably decreased by the inhibitors, we observed inhibition of NIK activity, as evidenced by reduced translocation of NF-κB2 and RelB into the nuclei (Fig. 4a, Supplementary Fig. 8b). Further analysis via immunofluorescence staining showed concurrently reduced expression of NF-κB2 and RelB in the nuclei of 1196PT as well as in TRAF3 knockout C and PT cell lines that were treated with 1 µM Amgen16, compared to that in cells treated with dimethyl sulfoxide (DMSO) (Fig. 4b, expanded panels in Supplementary Fig. 9). These results demonstrate that enhanced NF-κB signaling driven by PUM1-TRAF3 can be efficiently reverted by inhibiting NIK expression.

### PUM1-TRAF3 expression activates non-canonical NF-κB signaling via NF-κB2 activation

**Fig. 5:**
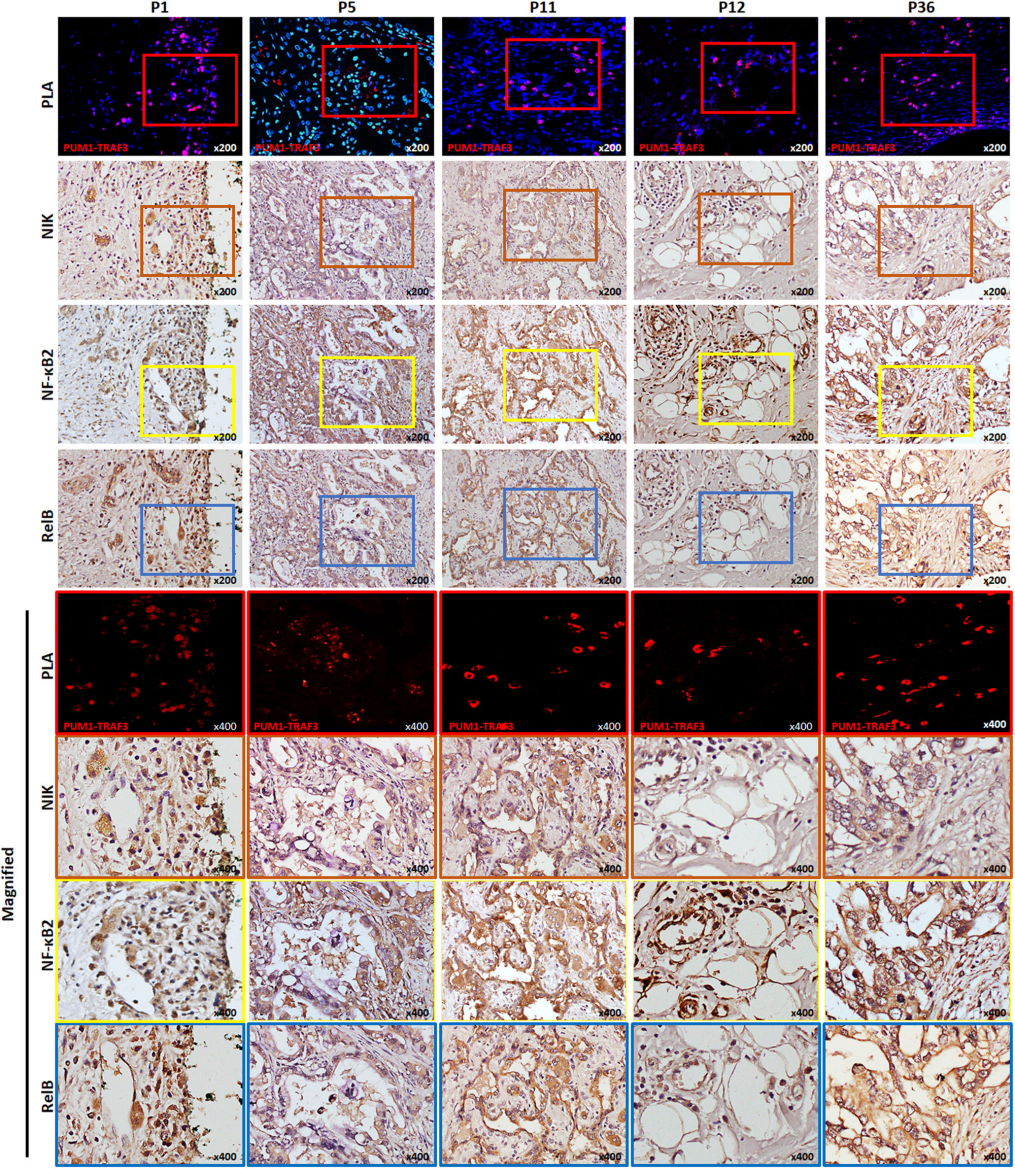
*PUM1-TRAF3* expression activates NIK and translocation of NF-κB2 and RelB. PLA was performed to visualize PUM1-TRAF3 protein expression in the P1, 5, 11, 12, and 36 patient tissue slides using PUM1 rabbit polyclonal antibody and TRAF3 mouse monoclonal antibody (PUM1-TRAF3, red; DAPI, blue), and elevated expression of NIK along with p52 and RelB in the nucleus could be observed as PLA signals expressing areas upon immunohistochemical analysis. Magnified areas are marked in red, orange, yellow, and blue for PLA, NIK, NF-κB2, and RelB, respectively.

Five patients’ tissue samples with PUM1-TRAF3 expression were confirmed using FISH with PUM1 and TRAF3 probes (P1, P5, P11, P12, and P36). These samples were further analyzed via in situ PLA to visualize the on-site gene expression. PLA was performed on patient tissue slides using a PUM1 rabbit polyclonal antibody and a TRAF3 mouse monoclonal antibody. To validate the specificity of the antibodies utilized in PLA, the signal was assessed using each antibody in combination with normal IgG or other antibodies including NF-κB2 and RelB and confirmed that the observed signals were specific to PUM1 and TRAF3 antibodies (Supplementary Fig. 10). Details of the antibodies employed in PLA can be found in Supplementary Table S3. Protein complexes were visualized in the indicated area of the slides (represented by spot-like PLA signals) (Fig. 5). Using immunohistochemical analysis, we visually observed the elevated expression of p52 and RelB in the nucleus as the area expressing the PUM1-TRAF3 fusion protein (Fig. 5, magnified marked area).

### Strong expression of NIK is associated with expression of PUM1-TRAF3 fusion and poor prognosis of BTC

**Fig. 6:**
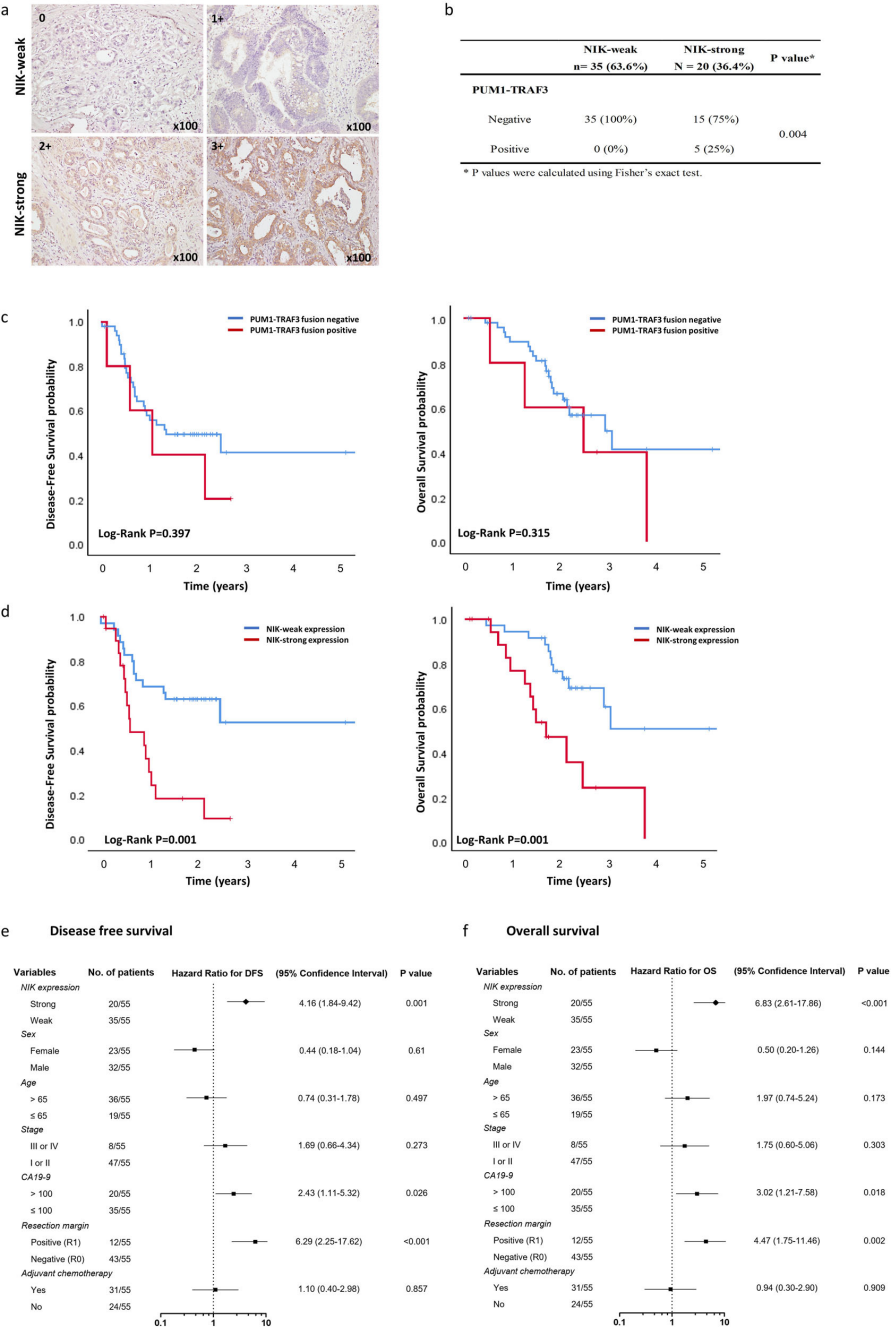
Expression of NIK is associated with expression of *PUM1-TRAF3* fusion and prognosis in patients with BTC. **a** Representative images of NIK expression by immunohistochemistry in patients with BTC. NIK. NIK staining was scored as an intensity of NIK-positive (+) tumor cells: no expression: 0, low expression: 1+, moderate expression: 2+, and strong expression: 3+. 0 or 1+ was considered as NIK-weak whereas 2+ or 3 + IHC staining scores of NIK was considered NIK-strong group. **b** Relative frequency of NIK-weak (*n* = 35) and NIK-strong (*n* = 20) groups according to the *PUM1-TRAF3* fusion status. **c** Kaplan–Meier analysis for survival according to *PUM1-TRAF3* fusion in patients with BTC (*n* = 55). Disease-free survival (DFS) and overall survival (OS) were not significantly different between fusion-negative (*n* = 50) and fusion-positive group (*n* = 5). **d** Kaplan–Meier analysis for survival according to NIK expression in patients with BTC (*n* = 55). NIK-weak group (*n* = 35) presented significantly prolonged survival in DFS and OS compared to NIK-strong group (*n* = 20). **e**, **f** Forest plots for multivariate Cox proportional hazards analysis of the contribution of clinical factors to DFS and OS presented NIK-strong expression was significantly associated with poorer DFS and OS.

Next, we evaluated the clinical implications of NIK expression in patients with BTC. IHC of the surgical samples classified 55 BTC patients into two groups: 35 (63.6%) as NIK-weak and 20 (36.4%) as NIK-strong, based on the intensity of NIK-positive (+) tumor cells (no expression, 0; low expression, 1+; moderate expression, 2+; and strong expression, 3+; NIK-weak = 0 or 1+, NIK-strong = 2+ or 3+) (Fig. 6a representative image of NIK IHC, Supplementary Table S6 for patient details). We found that NIK-strong was significantly over-represented in the PUM1-TRAF3 positive group (5/5 in PUM1-TRAF3 positive, 15/50 in PUM1-TRAF3 negative, *p* = 0.004) (Fig. 6b). Survival analysis showed that NIK expression (NIK-weak vs. NIK-strong) is a stronger factor for 2-year disease-free survival (DFS, 62.9% vs. 18.0%, log-rank *p* = 0.001) and overall survival (OS, 46.3% vs. 18.0%, log-rank *p* = 0.001) than PUM1-TRAF3 fusion (negative vs. positive; log-rank *p* = 0.397 and 0.315 for 2-year DFS and OS, respectively) (Fig. 6c, d). In addition, multivariate analysis via a Cox proportional hazards model identified strong NIK expression (and not the PUM1-TRAF3 fusion) as a prognostic marker of DFS (HR 4.16, 95% CI 1.84–9.42, *P* = 0.001) and OS (HR 6.83, 95% CI 2.61–17.86, *P* < 0.001) with two other variables: positive resection margin (HR 6.29, 95% CI 2.25–17.62, *P* < 0.001 in DFS; HR 4.47, 95% CI 1.75–11.46, *P* = 0.002 in OS) and higher serum CA19-9 levels (HR 2.43, 95% CI 1.11–5.32, *P* = 0.026 in DFS; HR 3.02, 95% CI 1.21–7.58, *P* = 0.018 in OS) (Fig. 6e, f). Altogether, these results indicated that NIK expression is associated with PUM1-TRAF3 fusion expression and poorer prognosis in patients with BTC.

## Discussion

BTC constitutes rare malignant tumors that are difficult to early diagnose and exhibit poor prognosis owing to the lack of markers. Thus, we analyzed BTC patient tissues via whole-transcriptome sequencing followed by a bioinformatics study and discovered a fusion gene PUM1-TRAF3 in BTC. Gene- and protein-level validation confirmed the presence of this gene and its recurrence in an extended cohort. We found that the expression of the fusion protein enhanced the proliferation, migration, invasion, and tumorigenesis abilities of BTC cells via constitutive activation of the non-canonical NF-κB pathway with NIK accumulation driven by rescuing NIK from degradation. Strong expression of NIK by IHC in cancer tissue was significantly associated with both PUM1-TRAF3 expression and poor prognosis of patients with BTC.

**Fig. 7:**
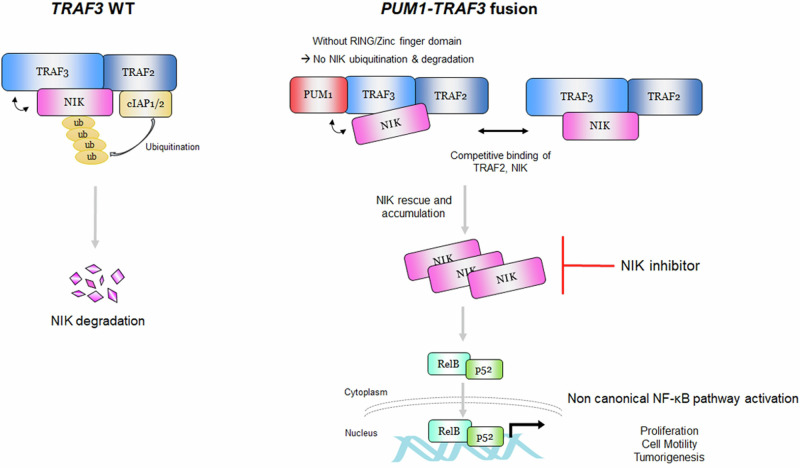
Action of PUM1-TRAF3 over TRAF3 in non-canonical NF-κB signaling. TRAF3 regulates NIK by binding and degrading it, whereas PUM1-TRAF3 binds to NIK but avoids degradation due to: 1) the lack of RING and zinc finger domains, and 2) reduction in TRAF2-TRAF3 complex formation by recruitment of TRAF2 by PUM1-TRAF3, thereby reducing its availability and rescuing NIK from degradation. NIK inhibitor can block the activation of the non-canonical NF-κB pathway by PUM1-TRAF3 expression.

Dysregulation of NF-κB signaling has been described in many cancers^23,24^. NF-κB signaling is activated when TNF receptor members (including B cell-activating factor, receptor activator of NF-κB, and CD40 ligand) induce the degradation of TRAF3. In an inactivated state, TRAF3 negatively regulates NIK by inducing NIK degradation to maintain low levels of NIK. When the signal is activated, NIK levels increase, which activates the phosphorylation of IKKα^13,17,18,25,26^. In turn, IKKα phosphorylates the inactive NF-κB p100 and RelB dimers. Activated p100 leads to the generation of p52, and nuclear translocation of RelB-containing p52 from the cytoplasm induces the expression of EMT genes^13^. Naïve TRAF3 physically binds NIK and consists of a RING finger, a zinc finger, and a MATH domain. Liao et al. reported that the N-terminal region of NIK interacts with the MATH domain of TRAF3 to mark NIK for degradation by proteasomes^25,27^. Furthermore, Sanjo et al. reported that the MATH domain is required for NIK binding, whereas the RING finger and zinc finger domains are required for ubiquitination and degradation of NIK, respectively^28^. In their study, the mutant TRAF3 that lacked RING finger and zinc finger domains was able to bind to NIK but was unable to participate in the constitutive ubiquitination and degradation of NIK^28^. He et al. suggested that both the RING finger and MATH domains are required for inhibition of the non-canonical NF-κB pathway; mutants of TRAF3 that lack either the RING finger or MATH domain fail to suppress NIK expression. While the MATH domain of PUM1-TRAF3 allows it to bind to NIK, a lack of the RING and zinc finger domains prevents PUM1-TRAF3 from acting in the degradation of NIK, which results in increased NIK levels. Alternatively, the increase in NIK levels may be due to a decrease in the binding of naïve TRAF3 and TRAF2. When TRAF2-TRAF3 binds to NIK, the cellular inhibitor of apoptosis protein (cIAP1/2) and the ubiquitin ligase activities of cIAP1/2 interact with NIK and mark NIK as a target for degradation by proteasomes^17,18,25,27,29^. He et al. reported that interactions between TRAF3 and TRAF2 are mediated by the TRAF-C domain, which is part of the MATH domain of TRAF3^17^. We noted that PUM1-TRAF3 bound to TRAF2 without changes in TRAF2 expression. Thus, competitive binding of PUM1-TRAF3 to TRAF2 over naïve TRAF3 may hinder formation of the TRAF3-TRAF2 complex required for NIK binding, thereby rescuing NIK from degradation (Fig. 7).

While further studies are required to determine whether the PUM1-TRAF3 fusion is a druggable target, we successfully showed that it significantly reduces the proliferation of BTC tumor cells, elicits a reduction in p52 expression in the nucleus, and suppresses NIK accumulation, by treating cells with the NIK inhibitor B022. Consistent with our hypothesis, Seubwai et al. also reported increased expression of NF-κB, including p52 subunits, in CCA patient tissues, and that inhibition of NF- κB expression reduces cell proliferation and tumor size in CCA-inoculated mice^30^. Another recent study also reported that TRAF3 inactivation induces the development of intrahepatic CCA through NIK activation^31^. Taken together, the results of our study and previous reports suggest that PUM1-TRAF3 inhibits TRAF3 by competitively binding TRAF2 and NIK, thereby inducing the relative inactivation of TRAF3 and resulting in CCA proliferation via the non-canonical NF-kB pathway.

For several years, pharmaceutical companies have sought to target the NF-κB pathway by repressing the DNA-binding activities of NF-κB, stabilizing IκB inhibitors with proteasome inhibitors, and inhibiting upstream IκB kinases^32^. However, severe toxicity and safety concerns associated with the therapeutic use of non-selective NF-κB inhibitors have prevented their clinical application^33^. More recently, research has focused on selective inhibition of the non-canonical NF-κB pathway by targeting IKKα or NIK^34^. However, IKKα inhibitors have limited specificity because IKKα is structurally homologous with IKKβ; IKKα forms a complex with IKKβ, and this complex plays a role in canonical NF-κB signaling. Thus, targeting NIK may be an improved method to achieve the inhibition of non-canonical NF-κB signaling. Currently, pharmaceutical companies are developing NIK inhibitors for multiple myeloma and prostate cancer^34–37^. These developments may also result in new treatment options for biliary cancers that express the PUM1-TRAF3 fusion gene.

Our study has some limitations. We experimentally identified the function of the *PUM1-TRAF3* fusion by inducing the accumulation of NIK via partial competition with TRAF3. Unfortunately, due to the lack of a specific PUM1-TRAF3 fusion protein antibody, we had to analyze PUM1-TRAF3 expression indirectly using PLA in patient tissues. The availability of a specific antibody to PUM1-TRAF3 could greatly enhance our understanding of its expression, not only in BTC but also in other types of cancer. We also statistically confirmed that overexpression of NIK was associated with a poor prognosis in patients with BTC, but the relationship between PUM1-TRAF3 fusion and patient survival could not be confirmed. This is possibly due to the small number of patients included in this study. Further study with a large number of patients is needed to determine the correlation between the fusion gene and prognosis of BTC.

In summary, we identified a PUM1-TRAF3 fusion gene expressed in Korean BTC patients. Expression of the fusion gene elicited the activation of non-canonical NF-κB signaling via NIK and resulted in BTC tumorigenesis; treatment with a NIK inhibitor reversed the PUM1-TRAF3-induced NF-κB activation and the proliferation of BTC cells. Fusion gene expression was significantly correlated with NIK expression, which was significantly associated with poor prognosis of patients with BTC. Accordingly, we suggest that the PUM1-TRAF3 fusion gene may have significant clinical implications as a therapeutic target in BTC.

## Methods

### Patients and sample preparation

Patient samples were obtained from individuals who had undergone surgery with a curative intent for BTC at the Severance Hospital, Yonsei University College of Medicine. The study protocol conformed with the ethical standards of the institutional research committee and the 1964 Helsinki declaration and its later amendments or comparable ethical standards. The Ethical Committee and Institutional Review Board of Yonsei University College of Medicine approved the protocol for tissue acquisition from the patient specimens (Institutional Review Board approval code: IRB 4-2011-0625, November 24, 2011) and tissues were collected after written informed consent was obtained from patients. Tumors were staged in accordance with the staging classification of the 7th edition of the American Joint Committee on Cancer (AJCC). Samples and clinical information were obtained from study-appropriate consenting individuals from the Severance Hospital, Yonsei University College of Medicine.

### RNA sequencing

Total RNA was extracted from paired-normal and cancer tissues from five BTC patients using RNeasy Miniprep kits (Qiagen, Valencia, CA, USA) (Supplementary Table S1). Subsequently complementary DNA (cDNA) was synthesized and subjected to end-repair and poly (A) addition and ligated with sequencing adapters using the TruSeq RNA Sample Prep Kit (Illumina, San Diego, CA, USA). Subsequent libraries were sequenced with an Illumina HiSeq2500 sequencer (Illumina) as recommended by the manufacturer. We generated, on an average, ~43 million paired-end (2 × 100-bp) reads per sample. Differentially expressed genes were visualized with the MeV microarray analysis platform (https://webmev.tm4.org /about).

### Fusion gene discovery

Candidate fusion genes were detected using ChimeraScan (version 0.4.5), FusionCatcher (version 0.99.4d), and JAFFA (version 1.06) against the hg19 human reference. In-house filtering steps were applied to discover gene fusion events (Fig. 1a). For ChimeraScan^38^, fusion partners with CDS (coding sequence) were selected, and to reduce false positives, candidate genes were required to have at least one spanning read mapped to the fusion junction. For FusionCatcher^39^, which is designed to yield a high real-time PCR validation rate, no further filtering processes were applied due to a few reported fusion events per sample. For JAFFA^40^, we selected fusions with high and moderate categories. Next, fusions detected in tumor samples were discarded with the following criteria: first, if they were found in pooled normal samples; second, if they were found in in-house curated fusion genes from RNA-seq of 462 lymphoblastoid cell line samples from five populations of the 1000 Genomes project (Geuvadis project)^41^ by using ChimeraScan with parameter r, 280 and public RNA-seq data (BodyMap provided by ChimeraScan authors and those of Greger and colleagues^42^. Among the remaining fusion genes, we only selected fusion genes detected by more than two tools. To identify and predict functional fusions, we searched for kinase, oncogene, tumor suppressor, and known bile duct cancer genes from candidate fusion partner genes. To annotate fusions, we used the COSMIC v77 fusion list, the Cancer Gene Census list, the Mitelman Database of Chromosome Aberrations and Gene Fusions in Cancer from ChimerDB3.0^43^, FusionCancer (a database of fusion genes in human cancers)^44^, fusionhub^45^, tumorfusion^46^, and a study by Gao et al.^47^, in which tumor fusions were detected among multiple cancers from TCGA. The final two candidate fusions were further validated by conventional PCR amplification, followed by Sanger sequencing.

### Fusion gene screening for TCGA cohorts and a BTC cohort

To screen for *PUM1-TRAF3* fusion expression in BTC and adjacent cancers, fusion analysis was performed using JAFFA with RNA-seq raw data (fastq) for TCGA-CHOL (*N* = 44; 36 tumors and 9 normal), TCGA-LIHC (*N* = 424; 374 tumors and 50 normal), and TCGA-PAAD (*N* = 183; 179 tumors and 4 normal). To screen for and analyze fusion in intrahepatic cholangiocarcinoma, we used RNA-seq raw data for seven paired samples of Caucasian intrahepatic cholangiocarcinoma (iCCA) from GSE63420^7^ with JAFFA. Graphical representation of fusion was achieved with Arriba (https://github.com/suhrig/arriba).

### PCR and droplet digital PCR (ddPCR)

Total RNA was extracted using the RNeasy Mini Kit (Qiagen), following the manufacturer’s instructions, and quantified using an ND-1000 Nanodrop spectrometer (NanoDrop Technologies, Wilmington, DE, USA). Reverse transcription was performed using Superscript II, RNaseOUT, oligo (dT) primer, and dNTPs (Invitrogen, Carlsbad, CA, USA). PCR was carried out using Platinum PCR SuperMix High Fidelity (Invitrogen), and PCR products were analyzed using agarose gel electrophoresis. ddPCR was performed following the manufacturer’s protocol. PCR samples were loaded on Bio-Rad’s QX100 droplet reader, and ddPCR data analysis was performed with ddPCR primers (PT2), QuantaSoft analysis software version 1.3.2.0, and the QX100 droplet reader. DNA was recovered by breaking oil droplets with chloroform, followed by centrifugation. The lower phase containing the PCR product was further analyzed via Sanger sequencing with ddPCR (PT2) primers. The primer sequences for PCR and ddPCR are listed in Supplementary Table S2.

### Cell culture

Human BTC cell lines SNU245, SNU308, SNU478, SNU869, SNU1079, and SNU1196 were obtained from the Korea Cell Line Bank (KCLB, Seoul, Korea). These cell lines were maintained in Roswell Park Memorial Institute medium (RPMI1640; Invitrogen) supplemented with 10% fetal bovine serum (FBS; Hyclone, Logan, UT, USA). Hucct-1, OZ, and KKU-100 cells were obtained from the Japanese Collection of Research Bioresources Cell Bank. These cell lines were maintained in Dulbecco’s modified Eagle’s medium (DMEM; Invitrogen) supplemented with 10% FBS. The 293FT cells were purchased from Invitrogen and maintained in DMEM supplemented with 10% FBS. Cells were maintained at 37 °C in a humidified incubator with 5% CO2. NIK inhibitors, Amgen16 and B022, were purchased from Sigma-Aldrich (St. Louis, MO, USA) and Selleck Chemicals (Houston, TX, USA), respectively.

### Gene cloning and generation of TRAF3 knockout cell lines

The fusion transcript sequence was amplified from a patient 1 (P1 or B01) cancer sample via nested PCR using the PUM1-TRAF3 primers PT3 and PT4. PCR products were eluted and inserted into the NotI site of the pLenti-c-mGFP vector (OriGene Technologies, Rockville, MD, USA) using In-Fusion Cloning kits (Takara). TRAF3 head and tail sequences were cloned into the EcoRI and NotI site of the pReceiver-LV122 vector (GeneCopoeia, Rockville, MD, USA). Primer sequences are listed in Supplementary Table S2. Lentiviral vector and packaging vectors (OriGene) were co-transfected into 293FT cells using Lipofectamine 2000 (Invitrogen); the supernatant was collected and added to the BTC cell lines to transduce the PT protein. Green fluorescent protein-labeled cells were selected using flow cytometry analysis. The SNU1196 cell line was used to generate TRAF3 knockout cells using the CRISPR/Cas9 genome-editing technology designed to target exon 2 (gRNA sequence: 5′-AATGGAGTCGAGTAAAAAGATGG-3′) by Macrogen (Macrogen Korea, Seoul, Korea). Cells transfected with the CRISPR construct were selected using puromycin, and protein expression in colonies originating from single cells was further analyzed via western blotting.

### Western blotting and immunoprecipitation

Cells were lysed in a buffer containing 70 mM glycerophosphate (pH 7.2), 0.6 mM Na vanadate, 2 mM MgCl2, 1 mM ethylene glycol tetraacetic acid (EGTA), 1 mM dithiothreitol, 0.5% Triton X-100, 0.2 mM phenylmethylsulfonyl fluoride, and 1× complete protease inhibitor (Roche Applied Science, Nutley, NJ, USA). Proteins were separated on sodium dodecyl sulfate polyacrylamide gels and transferred onto a polyvinylidene difluoride membrane (Immobilon-P; Millipore, Bedford, MA, USA), which was blocked with 5% (w/v) non-fat dry milk (Bio-Rad Laboratories, Hercules, CA, USA). The membranes were then incubated overnight at 4 °C with primary antibodies diluted at a ratio of 1:1000. A horseradish peroxidase-conjugated secondary antibody was used for final staining, and immunoblots were developed with the West Pico Chemiluminescent substrate (Thermo Fisher Scientific, Waltham, MA, USA). All blots derived from the same experiment were processed in parallel and the uncropped scans of important blots were shown in Supplementary Fig. 11. Western blot band intensity was analyzed using ImageJ software (https://imagej.nih.gov/ij/). The antibodies used for western blot analysis are listed in Supplementary Table S3.

### Proliferation assays

Cells were detached and plated in triplicate, at a density of 2 × 103 cells/well, in 96-well plates in 100 µl of complete medium. Twenty-four hours after plating, WST-8 (2-(2-methoxy-4-nitrophenyl)-3-(4-nitrophenyl)-5-(2,4-disulfophenyl)-2H-tetrazolium) (EZ-Cytox, Daeil Lab, Seoul, Korea) was added and incubated for 2 h. The absorbance at 450 nm was measured with a microplate reader.

### Flow cytometry

Cells were detached using Accutase (Sigma) and 1 × 106 cells were resuspended in 100 μL wash buffer containing 1%BSA and 2 mM EDTA in PBS. Cells were incubated with the antibodies for 1 hour, washed twice, and analyzed by FACS in an LSRII. Events were collected on the FSC/SSC dot plot and read on a CD133^+^ versus GFP^+^ dot plot. The gating strategy and antibodies used are shown in the Supplementary File (Supplementary Fig. S6e, Supplementary Table 3). All data were analyzed using FlowJo (BD).

### Tumorigenicity assay

For the in vivo tumorigenicity assay, all animal studies were conducted using protocols approved by the Institutional Animal Care and Use Committee. Experiments were carried out using 6-week-old male BALB/c nude mice (Japan SLC, Yokohama, Japan) and SNU1196 cells or TRAF3 knockout SNU1196 cells expressing PT (1196PT) and control vector (1196C) were subcutaneously injected into mice (5 × 10^6^ cells/site). Tumor dimensions were measured twice weekly, and tumor volume was calculated using the formula: (length × width × width)/2. The maximal total volume for all tumors in mice, permitted by ethical protocol, was 2000 mm^3^. The mice were sacrificed by CO2 inhalation and tumors were harvested. Tissues were collected after 4 weeks. Mice were euthanized by CO2 inhalation at the end of the experiment.

### Fluorescence in situ hybridization (FISH)

Patient formalin-fixed paraffin-embedded (FFPE) slides underwent FISH analysis following the SwiftFISH (Empire Genomics, Buffalo, NY, USA) protocol. Briefly, deparaffinized slides were incubated with pepsin, washed, and dehydrated, after which the probe mixture was added to the slides. The FISH probe consisted of two respective bacterial artificial chromosome probes for the PUM1 region (284 kb, labeled red) and TRAF3 region (309 kb, labeled green) and was mixed with SwiftFISH Buffer (Empire Genomics). Tissue slides and probes were denatured, hybridized, and counterstained with 4′,6-diamidino-2-phenylindole stain (DAPI). Fluorescence images were obtained with a Zeiss LSM 700 confocal microscope (Carl Zeiss, Berlin, Germany).

### In situ proximity ligation assay (PLA)

Deparaffinized slides were incubated with a rabbit polyclonal antibody against PUM1 (Santa Cruz Biotechnology, Dallas, TX, USA) and a mouse monoclonal antibody against TRAF3 (Santa Cruz) in an antibody diluent (DakoCytomation California, Inc., Carpinteria, CA, USA). After washing, PLA was performed using Duolink® In Situ Red Starter Kits (Sigma-Aldrich), according to the manufacturer’s protocol, to visualize the PUM1 and TRAF3 fusion proteins. Nuclei were labeled with DAPI, and fluorescence images were obtained using a Zeiss LSM 700 confocal microscope (Carl Zeiss).

### Immunohistochemical and immunofluorescence staining

Tissue slides were deparaffinized in xylene and rehydrated in graded alcohol. Endogenous peroxidase activity was blocked with 0.3% (v/v) hydrogen peroxide in methanol. Antigen retrieval was performed by microwaving the slides in a sodium citrate buffer (0.01 M, pH 6.0) for 5 min. To block nonspecific staining, sections were incubated with 10% (v/v) normal donkey serum for 1 h; then, the sections were incubated with appropriate antibodies overnight at 4 °C. Subsequent reactions were performed using Envision kits (DakoCytomation) following the manufacturer’s instructions. Immunoreactions were developed with the DAunfKO liquid diaminobenzidine substrate-chromogen system (DAB+) and counterstained with Harris hematoxylin (Sigma-Aldrich). The reaction was subsequently carried out with an LSAB+ Kit (DAKO), and sections were counterstained with Mayer’s hematoxylin, dehydrated, and observed under a BX51 microscope (Olympus, Tokyo, Japan). For immunofluorescence staining, tissue slides were visualized using Cy5-goat anti-rabbit immunoglobulin G antibody (IgG) dissolved in an antibody diluent and incubated for 30 min at room temperature. Between each step, three washing steps of 5 min each were performed on a rocking platform using phosphate-buffered solution (PBS). The slides were cover-slipped using mounting medium for observing fluorescence with DAPI (Vecta shield H-1200; Vector Laboratories, Inc. Burlingame, CA, USA). The pathologic scoring for NIK immunohistochemical (IHC) staining was independently conducted by the main researchers, J.H. Jo and D.E. Jung, who were blinded to patient information and the type of treatment. Patient slides were carefully reviewed and scored based on the intensity of NIK expression, categorized as follows: no expression (0), low expression (light brown, 1+), moderate expression (brown, 2+), and strong expression (dark brown, 3+). The antibodies used for immunohistochemical and immunofluorescence staining are listed in Supplementary Table S3.

### Statistical analysis

Data were analyzed using the χ2 and Fisher’s exact tests for categorical data and Student’s *t* test and Mann–Whitney U test for continuous variables. Multivariate analysis was performed to evaluate potentially significant factors in consideration of the influence of confounding clinical variables. Hazard ratio (HR), 95% confidence interval (CI), and *p*-values in multivariate analysis were calculated with a Cox proportional hazards model for disease-free survival (DFS) and overall survival (OS). Survival was estimated and compared using Kaplan–Meier analysis with the log-rank test. All statistical analyses were performed using IBM SPSS Statistics for Windows, version 25.0 (IBM Corp., Armonk, NY, US). A value of *p* < 0.05 was considered statistically significant.

### Supplementary information

Supplementery File

## Data Availability

The data in this study are openly available in the Gene Expression Omnibus under accession number GSE85589 (www.ncbi.nlm.nih.gov/geo) and BioProject database under accession PRJNA851994 (www.ncbi.nlm.nih.gov/sra).

## Code availability

Custom code was not used in this study. Public software was executed with default settings unless otherwise specified in the Methods section.
