# Supplementary material for: PUM1-TRAF3 fusion protein activates non-canonical NF-κB signaling via rescued NIK in biliary tract cancer
Source: NPJ Precis Oncol. 2024 Aug 1;8:170. doi: 10.1038/s41698-024-00654-2 (PMC11294552; doi:10.1038/s41698-024-00654-2)
Supplement: Supplementary file 1 — Supplementery File [file 41698_2024_654_MOESM1_ESM.pdf]

a

b

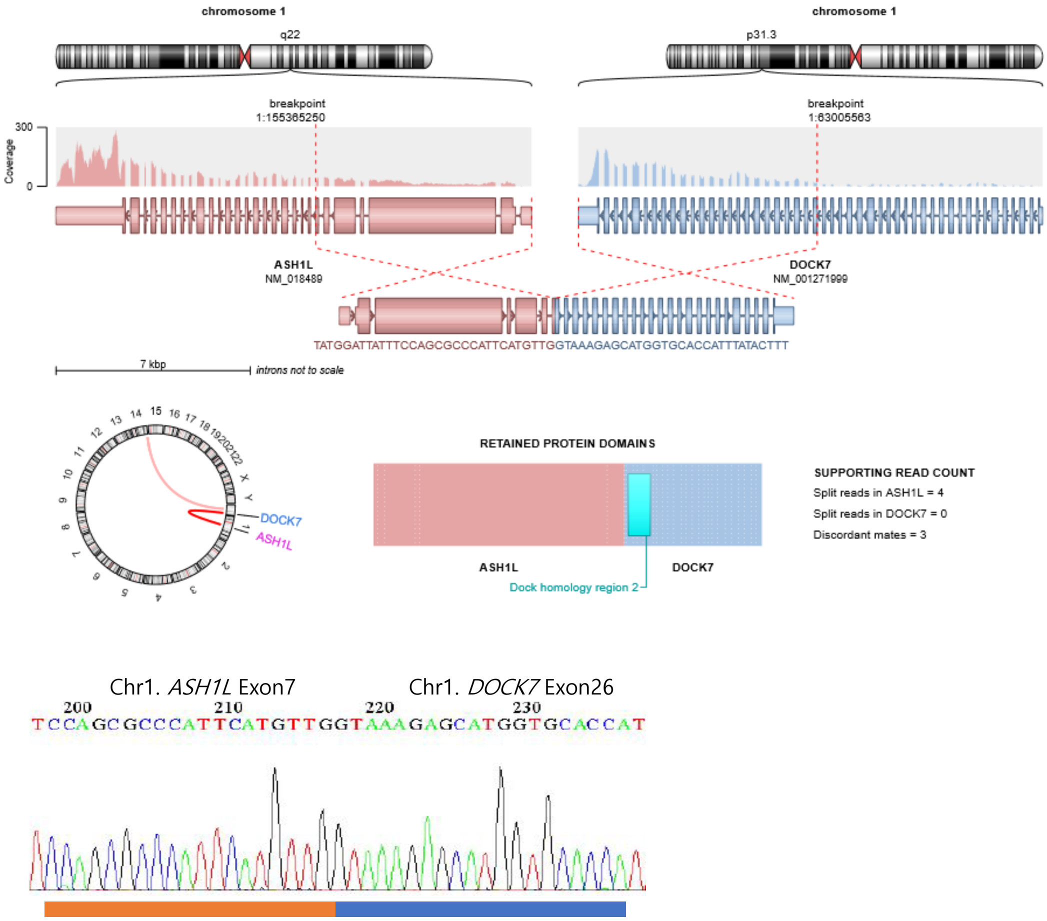

Supplementary Fig. 1. (a) Schematic representation of the *ASH1L-DOCK7* fusion involving *ASH1L* and *DOCK7* (both on chromosome 1). Schematic diagrams and Circos plots were visualized using Arriba.
(b) Confirmation of the chimeric junction between *ASH1L* and *DOCK7* in the index patient via Sanger sequencing.

Pum1-Traf3 transcript

atgagcgttgcatgtgtcttgaagagaaaagcagtgctttggcaggactctttcagcccccacctgaaacatcaccctcaagaaccagctaat
cccaacatgcctgttgttttgacatctggaacagggtcgcaagcgcagccacaaccagctgcaaatcaggctcttgcagctgggactcactc
cagccctgtcccaggatctataggagttgcaggccgttcccaggacgacgctatggtggactacttctttcagaggcagcatggtgagcag
cttggggga ggaggaagtggaggaggcggctataataatagcaaacatcgatggcctactggggataacattcatgcagaacatcaggt
gcgttccatggatgaactgaatcatgattttcaagcacttgctctggagggaagagcgatgggagagcagctcttgccaggtaaaaagtttt
gggaaacagatgaatccagcaaagatggaccaaaaggaatattcctgggtgatcaatggcgagacagtgcctggggaacatcagatcat
tcagtttcccagccaatcatggtgcagagaagacctggtcagagtttccatgtgaacagtgaggtcaattctgtactgtccccacgatcggag
agtgggggactaggcgttagcatggtggagtatgtgttgagctcatccccgggcgattcctgtctaagaaaaggaggatttggcccaagg
gatgcagacagtgatgaaaacgacaaaggtgaaaagaagaacaagggtacgtttgatggagataagctaggagatttgaaggaggag
ggtgatgtgatggacaagaccaatggtttaccagtgcagaatgggattgatgcagacgtcaaagattttagccgtacccctggtaattgcca
gaactctgctaatgaagtggatcttctgggtccaaaccagaatggttctgagggcttagcccagctgaccagcaccaatggtgccaagcctg
tggaggatttctccaacatggagtcccagagtgtccccttggaccccatggaacatgtgggcatggagcctcttcagtttgattattcaggca
cgcaggtacctgtggactcagcagcagcaactgtgggactttttgactacaattctcaacaacagctgttccaaagacctaatgcgcttgctgt
ccagcagttgacagctgctcagcagcagcagtatgcactggcagctgctcatcagccgcacatcggtttagctcccgctgcgtttgtccccaa
tccatacatcatcagcgctgctcccccagggacggaccccctacacagctggattggctgcagcagcgacactaggcccagctgtggtccctc
accagtattatggagttactccctggggagtctaccctgccagtcttttccagcagcaagctgccgctgccgctgcagcaactaattcagctaa
tcaacagaccaccccacaggctcagcaaggacagcagcaggttctccgtggaggagccagccaacgtcctttgaccccaaaccagaacca
gcagggacagcaaacggatccccttgtggcagctgcagcagtgaattctgcccttgcatttggacaaggtctggcagcaggcatgccagg
ttatccggtgttggctcctgctgcttactatgaccaaactggtgcccttgtagtgaatgcaggcgcgagaaatggtcttggagctcctgttcga
cttgtagctcctgccccagtcatcattagttcctcagctgcacaagcagctgttgcagcagccgcagcttcagcaaatggagcagctggtggt
cttgctggaacaacaaatggaccatttcgccctttaggaacacagcagcctcagccccagccccagcagcagcccaataacaacctggcatc
cagttctttctacggcaacaactctctgaacagcaattcacagagcagctccctcttctcccagggctctgcccagcctgccaacacatccttgg
gattcggaagtagcagttctctcggcgccaccctgggatccgcccttggagggtttggaacagcagttgcaaactccaacactggcagtgg
ctcccgccgtgactccctgactggcagcagtgacctttataagaggacatcgagcagcttgacccccattggacacagtttttataacggcctt
agcttttcctcctctcctggacccgtgggcatgcctctccctagtcagggaccaggacattcacagacaccacctccttccctctcttcacatgga
tcctcttcaagcttaaacctgggaggactcacgaatggcagtggaagatacatctctgctgctccaggcgctgaagccaagtaccgcagtgc
aagcagcgcctccagcctcttcagcccgagcagcactcttttctcttcctctcgtttgcgatatggaatgtctgatgtcatgccttctggcagga
gcaggcttttggaagattttcgaaacaaccggtaccccaatttacaactgcgggagattgctggacatataatggaattttcccaagaccagc
atgggtccagattcattcagctgaaactggagcgtgccacaccagctgagcgccagcttgtcttcaatgaaatcctccaggctgcctaccaac
tcatggtggatgtgtttggtaattacgtcattcagaagttctttgaatttggcagtcttgaacagaagctggctttggcagaacggattcgagg
ccacgtcctgtcattggcactacagatgtatggctgccgtgttatccagaaagctcttgagtttattccttcagaccagcagaatgagatggttc
gggaactagatggccatgtcttgaagtgtgtgaaagatcagaatggcaatcacgtggttcagaaatgcattgaatgtgtacagccccagtct
ttgcaatttatcatcgatgcgtttaaggg**acag**cgagtgatagacagccaagcagagaaactgaaggagcttgacaaggagatccggccc
ttccggcagaactgggaggaagcagacagcatgaagagcagcgtggagtccctccagaaccgcgtgaccgagctggagagcgtggac
aagagcgcggggcaagtggctcggaacacaggcctgctggagtcccagctgagccggcatgaccagatgctgagtgtgcacgacatcc
gcctagccgacatggacctgcgcttccaggtcctggagaccgccagctacaatggagtgctcatctggaagattcgcgactacaagcggc
ggaagcaggaggccgtcatggggaagaccctgtccctttacagccagcctttctacactggttactttggctataagatgtgtgccagggtct
acctgaacggggacgggatggggaaggggacgcacttgtcgctgtttttgtcatcatgcgtggagaatatgatgccctgcttccttggccg
tttaagcagaaagtgacactcatgctgatggatcaggggtcctctcgacgtcatttgggagatgcattcaagcccgaccccaacagcagcag
cttcaagaagcccactggagagatgaatatcgcctctggctgcccagtctttgtggcccaaactgttctagaaaatgggacatatattaaaga
tgatacaatttttattaaagtcatagtggatacttcggatctgcccgatccc

-**bold**, overlapping sequences

Supplementary Fig. 2. Chimeric junction between the *PUM1* and *TRAF3* genes in the *PUM1-TRAF3* fusion gene.

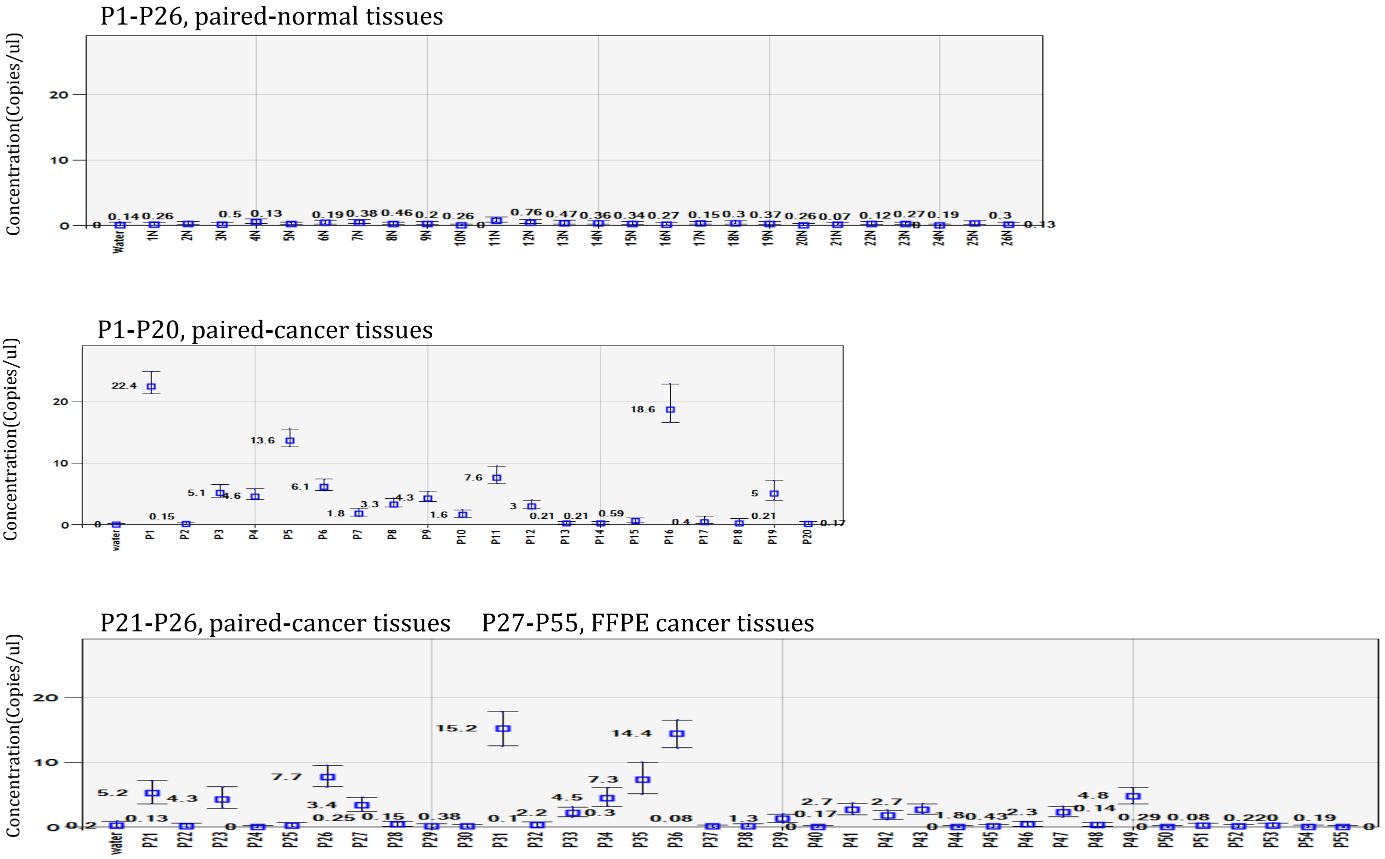

Supplementary Fig. 3. Droplet digital PCR (ddPCR) analysis of frozen tissues (P1-P26; Lane 1, P1N-P26N, paired normal; Lane 2, P1-P20, paired cancer; Lane 3, P21-P26, paired cancer) and FFPE samples (P27-P55; Lane 3, P27-55, FFPE cancer only).

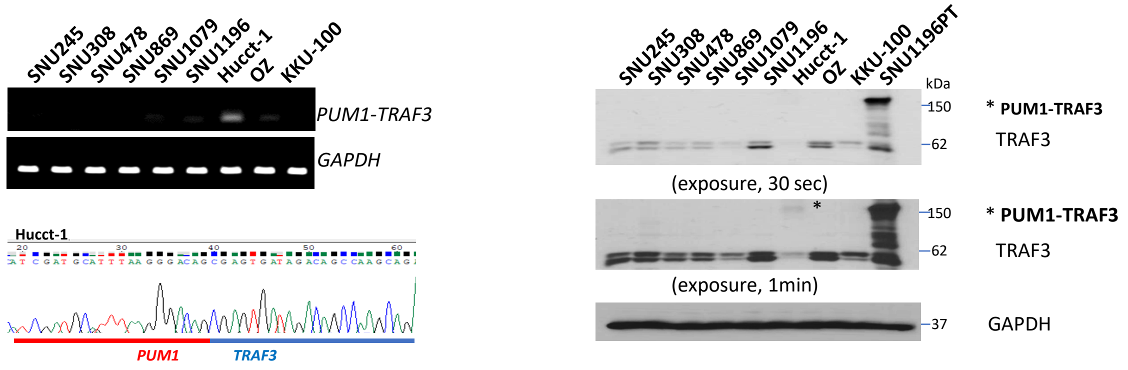

Supplementary Fig. 4. *Pum1-TRAF3* gene and protein expression in BTC cell lines confirmed via PCR and western blotting.

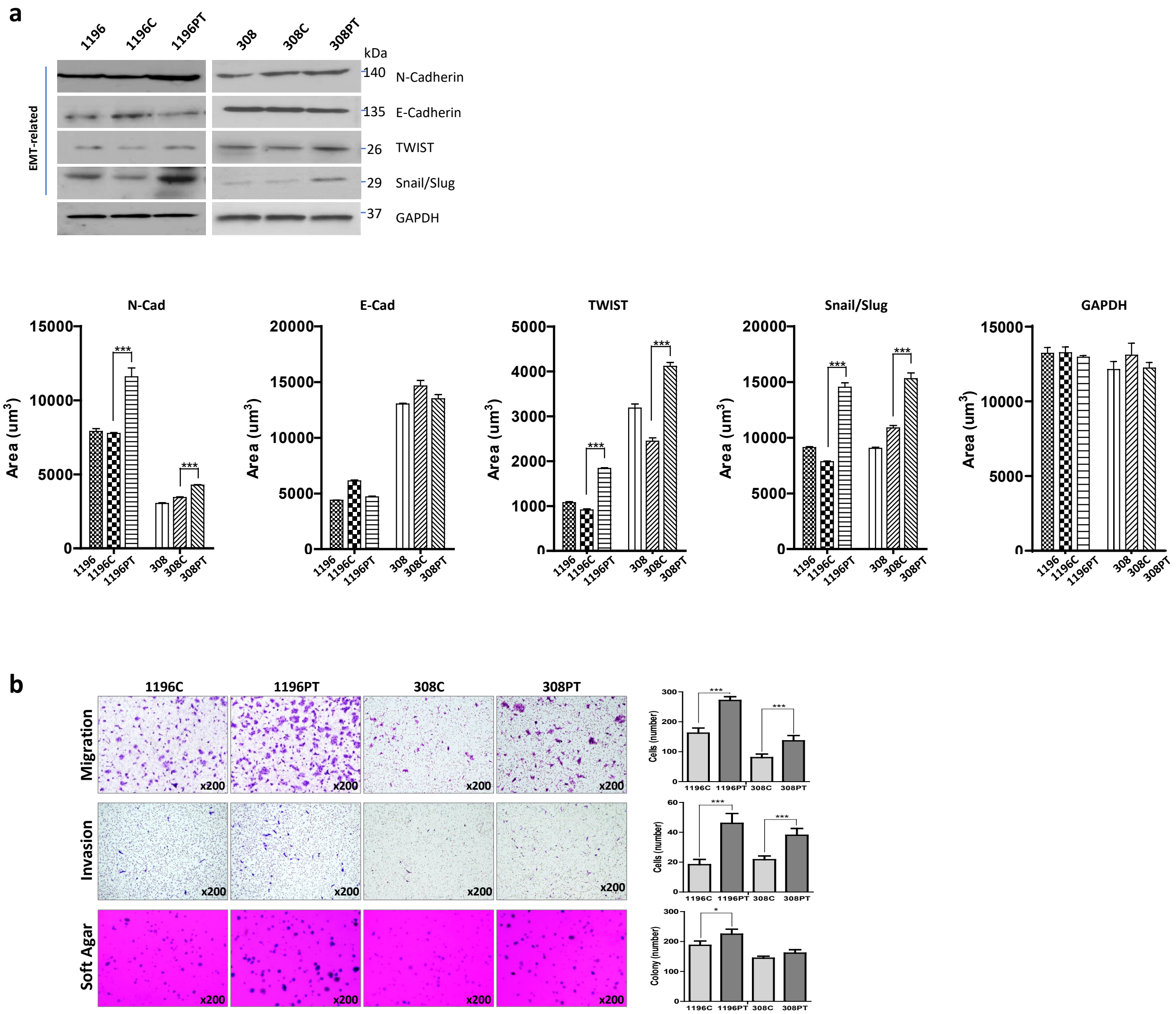

Supplementary Fig. 5. (a) Proteins differentially expressed in PUM1-TRAF3-transducing cells were analyzed by area, including epithelial-to-mesenchymal transition-associated proteins N-cadherin, E-cadherin, TWIST, and Snail/Slug (***, P<0.001). (b) Cell migration, invasion, and soft agar analysis in SNU1196 and SNU308 cells expressing Pum1-TRAF3 (*, P<0.05; ***, P<0.001).

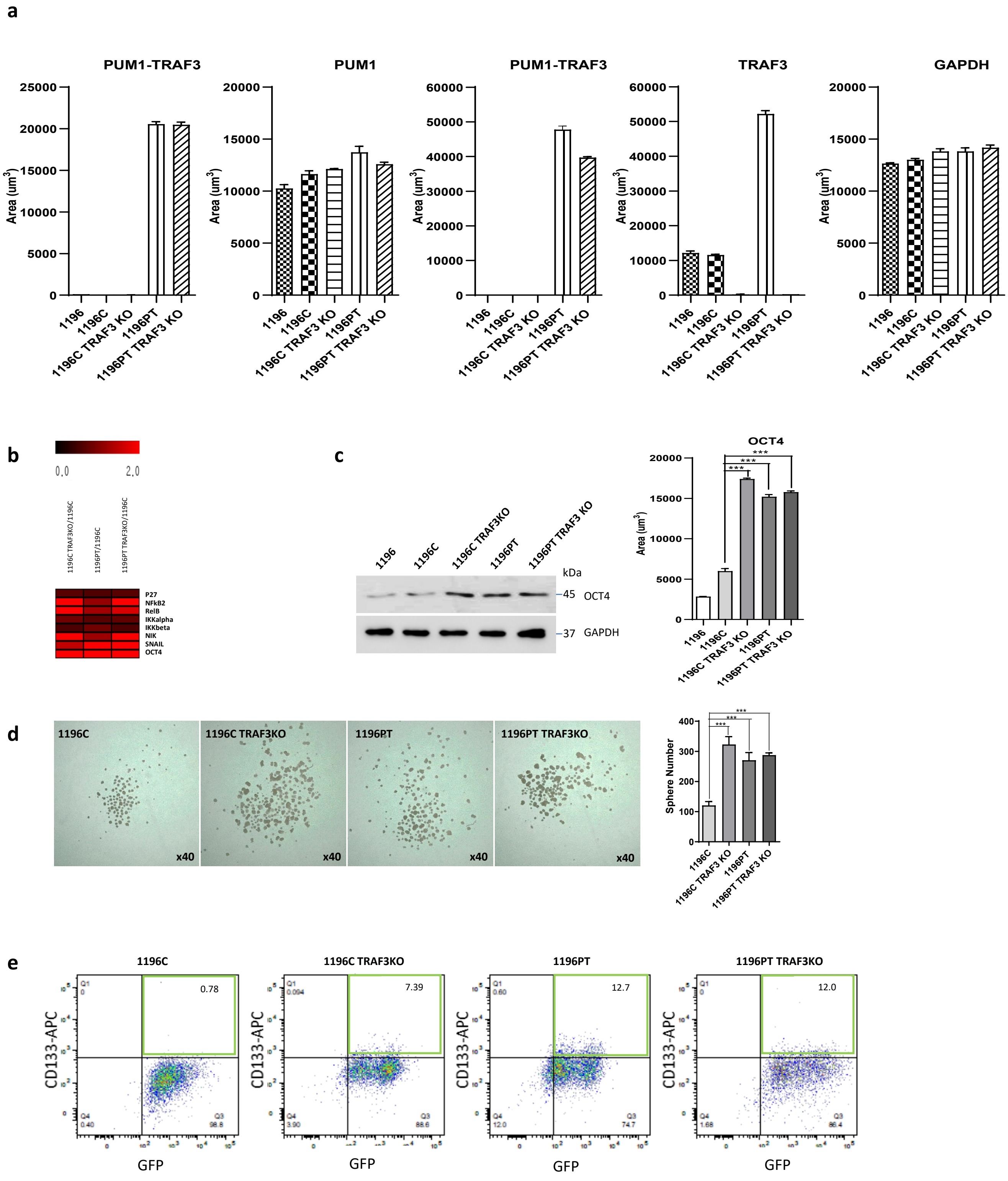

Supplementary Fig. 6. (a, c) Proteins differentially expressed in PUM1-TRAF3-transducing cells with or without TRAF3 expression, including PUM1, TRAF3, NIK, and OCT4, were analyzed by area (***, P<0.001). (b) Differentially expressed genes in PUM1-TRAF3-transduced cells by RNA sequencing. (d) Sphere formation assay and (e) CD133+ cell analysis by FACs to analyze changes in stemness (***, P<0.001).

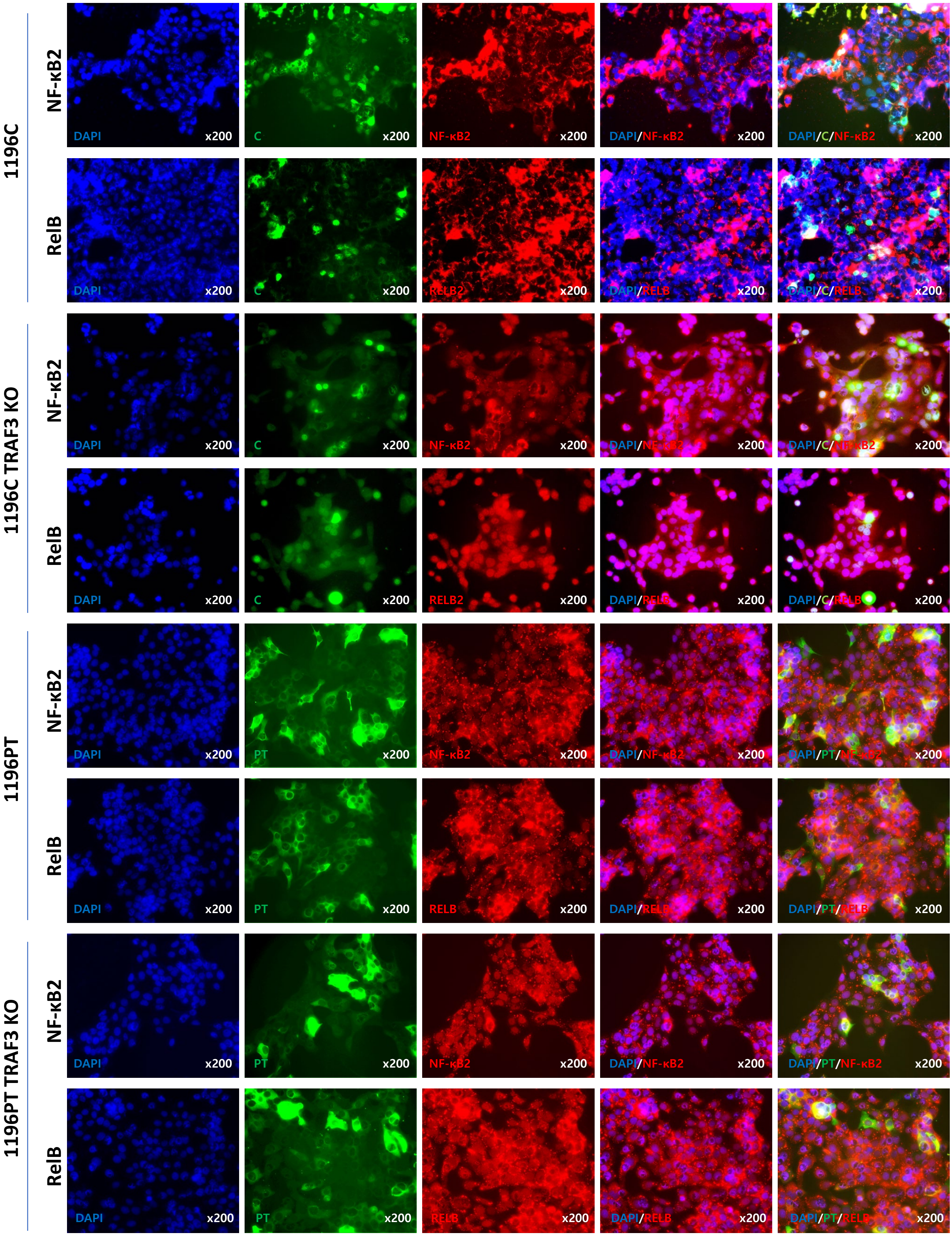

Supplementary Fig. 7. Immunofluorescence staining for NF-κB2 and RelB in SNU1196 control vector-transduced and SNU1196 PUM1-TRAF3-transduced cells with or without *TRAF3* expression. (DAPI, blue; Green, PT or C; Red, NF-κB2 or RelB).

a

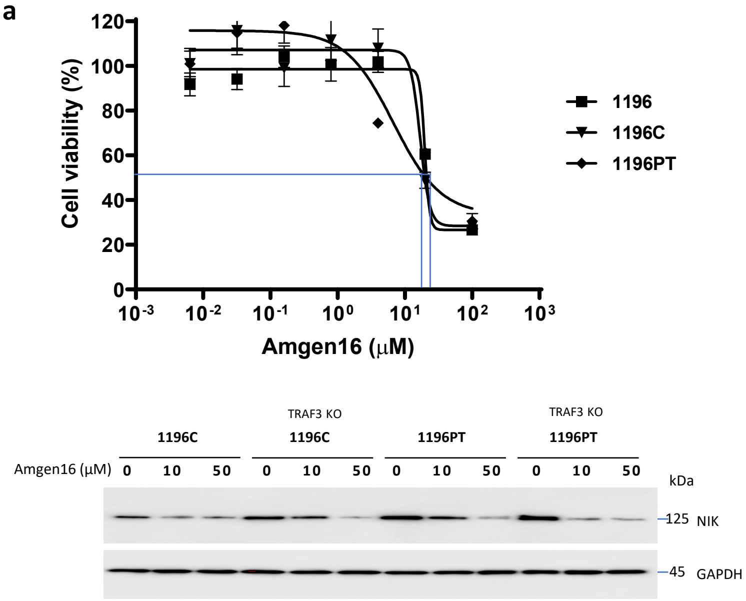

b

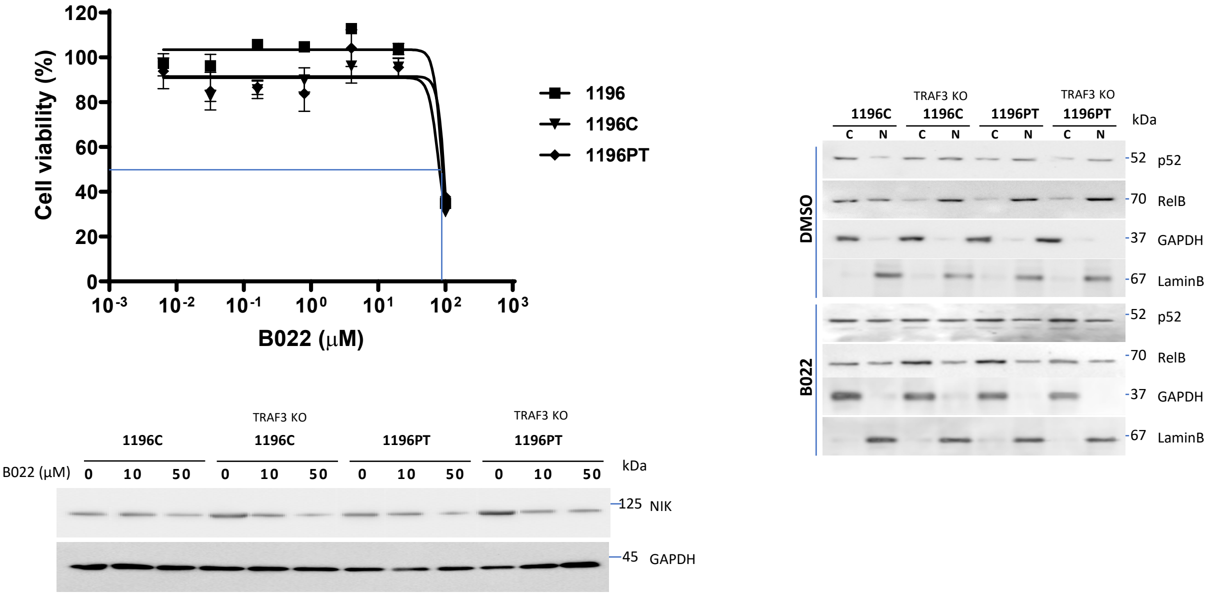

Supplementary Fig. 8. Cytotoxicity of NIK inhibitors (a) Amgen16 and (b) B022 and changes in NIK expression. B022 inhibits the translocation of NF-κB2 and RelB (C, cytoplasmic fraction; N, nucleic fraction).

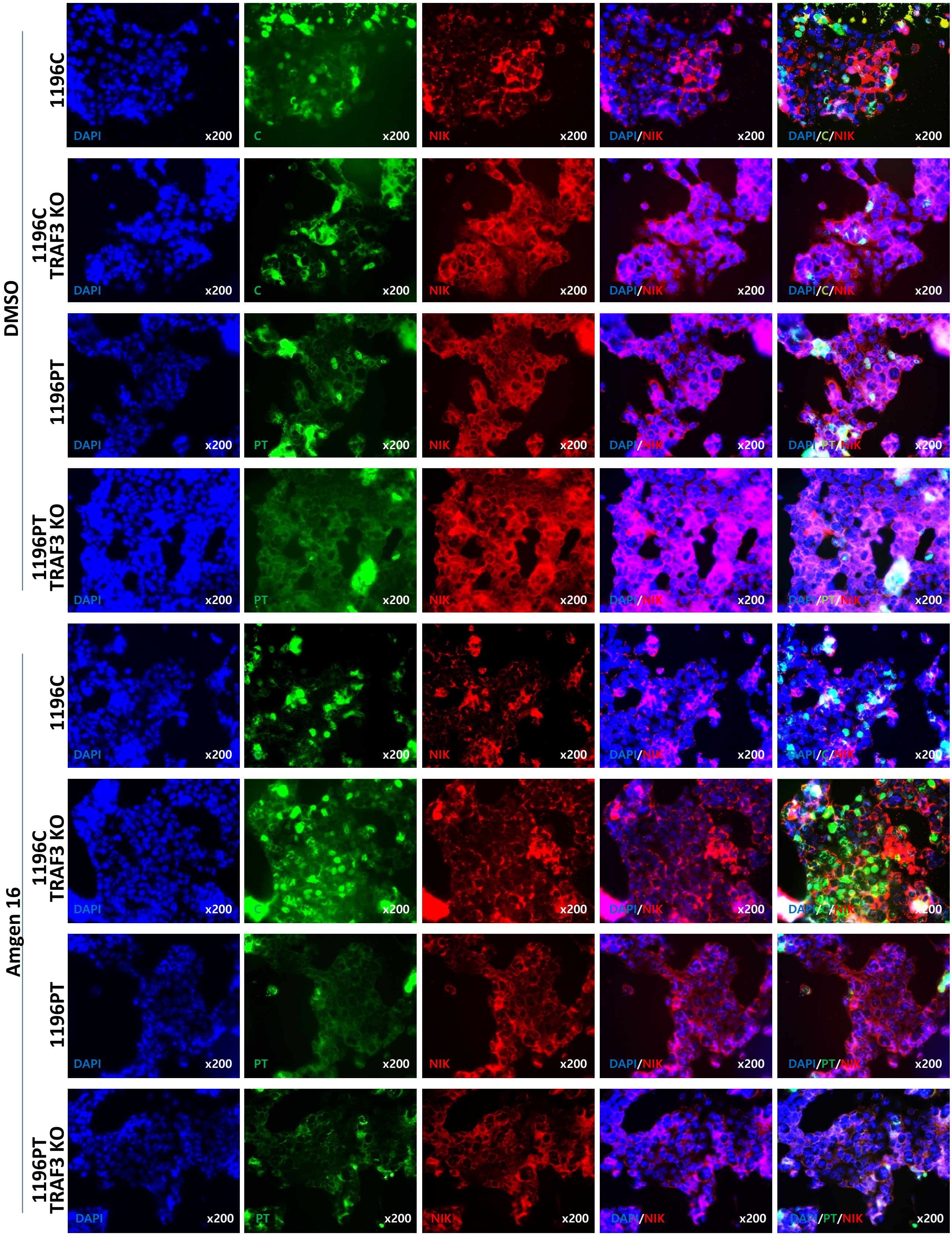
DMSO
Amgen 16
1196C
1196C TRAF3 KO
1196PT
1196PT TRAF3 KO
DAPI
C
PT
NIK
DAPI/NIK
DAPI/C/NIK
DAPI/PT/NIK
x200

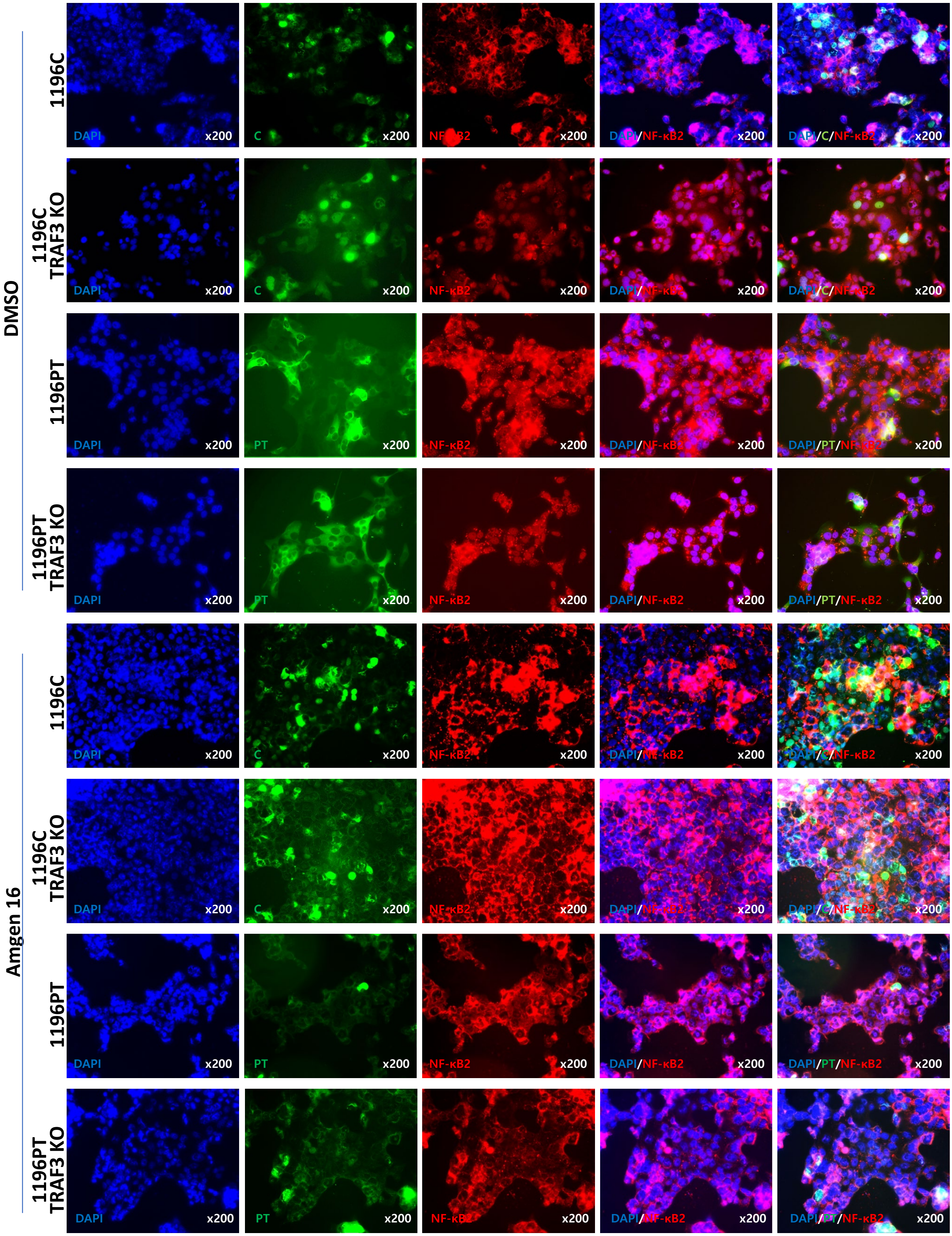
DMSO
Amgen 16
1196C
1196C TRAF3 KO
1196PT
1196PT TRAF3 KO
DAPI
C
PT
NF-κB2
DAPI/NF-κB2
DAPI/C/NF-κB2
DAPI/PT/NF-κB2
x200

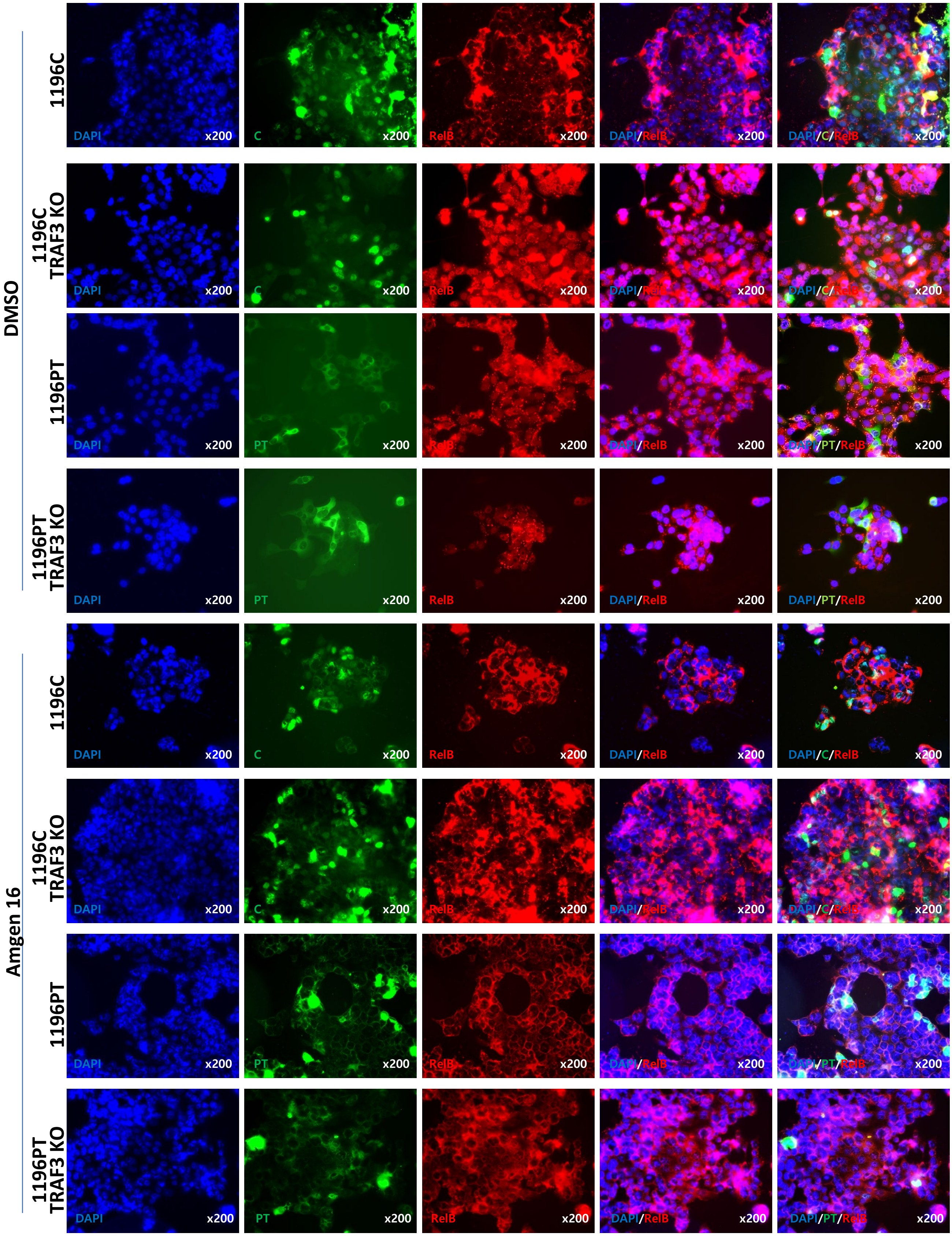

Supplementary Fig. 9. PUM1-TRAF3-induced NF-κB activation results in the translocation of NF-κB2 and RelB from the cytoplasm to the nucleus. (DAPI, blue; Green, PUM1-TRAF3 or control vector; Red, NF-κB2 or RelB).

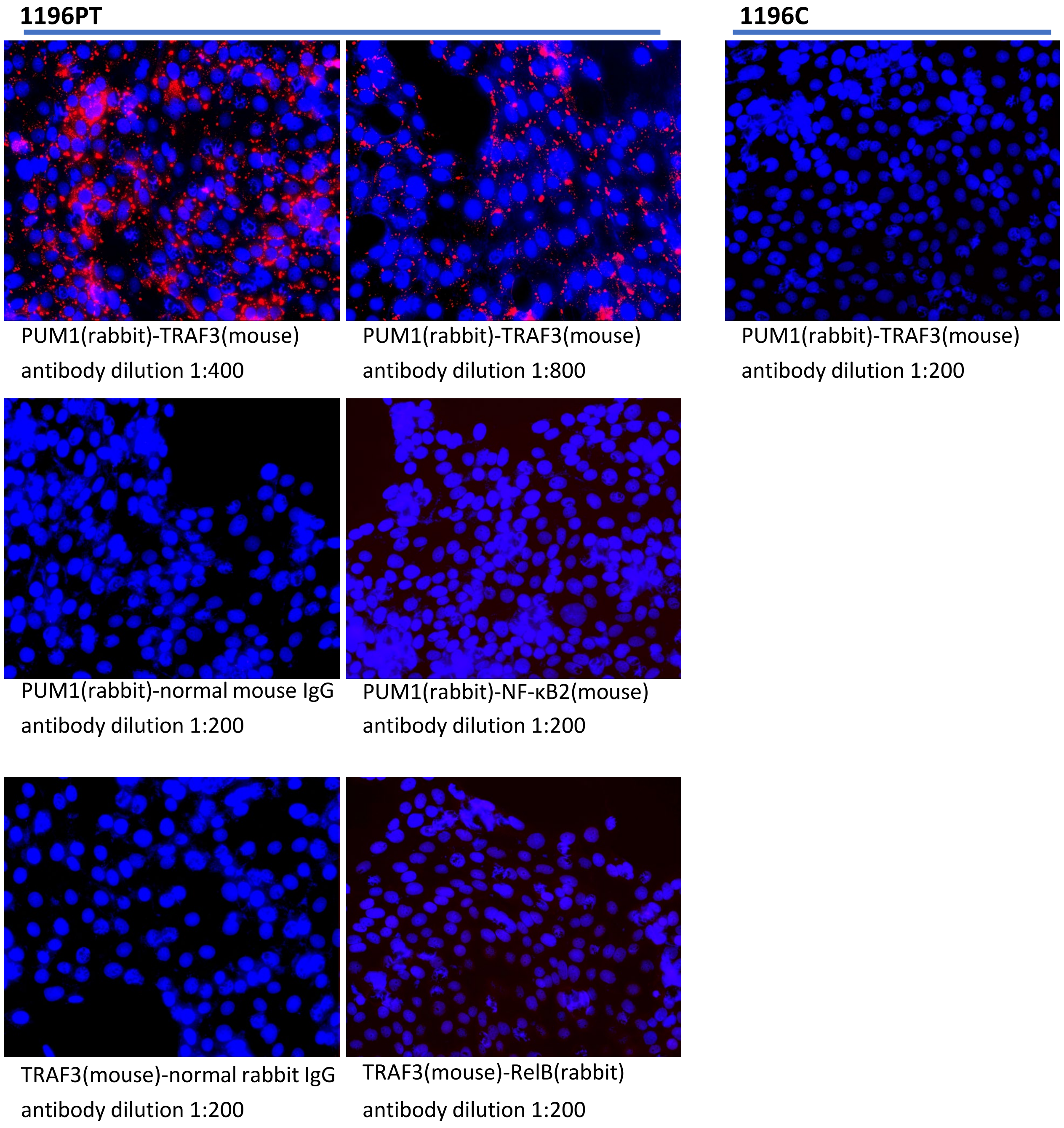

Supplementary Fig. 10. Antibody specificities utilized in proximal ligation assay.

2e

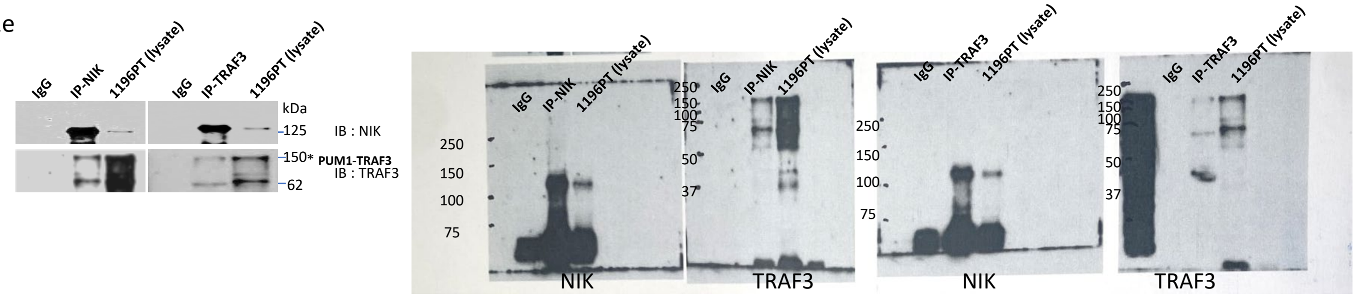
IgG
IP-NIK
1196PT (lysate)
IgG
IP-TRAF3
1196PT (lysate)
kDa
125
IB : NIK
150*
PUM1-TRAF3
IB : TRAF3
62
250
150
100
75
50
37
NIK
TRAF3
NIK
TRAF3

2f

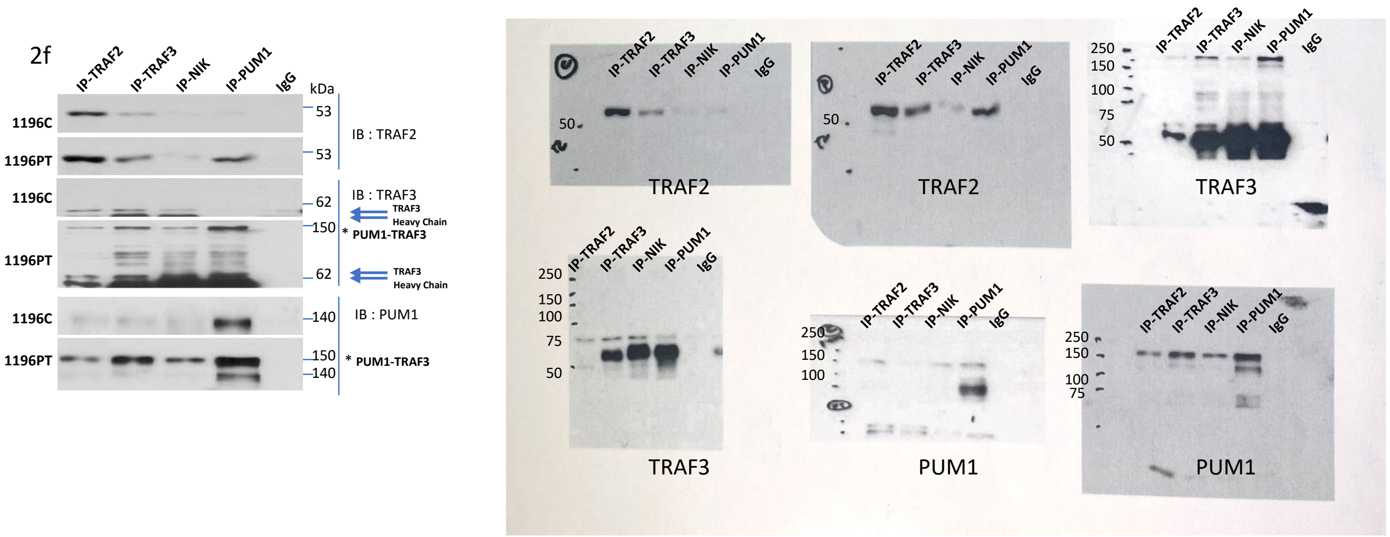
IP-TRAF2
IP-TRAF3
IP-NIK
IP-PUM1
IgG
kDa
1196C
1196PT
53
IB : TRAF2
62
IB : TRAF3
TRAF3 Heavy Chain
150
* PUM1-TRAF3
140
IB : PUM1
250
100
75
50
TRAF2
TRAF3
PUM1

2h

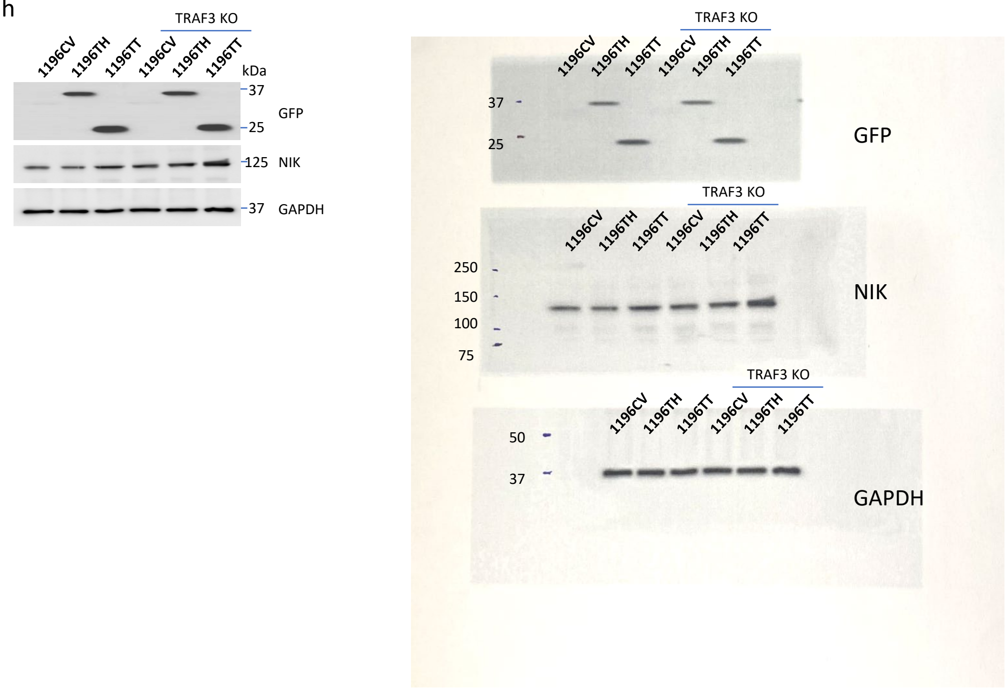
TRAF3 KO
1196CV
1196TH
1196TT
kDa
37
25
GFP
125
NIK
GAPDH
250
150
100
75
50

2i

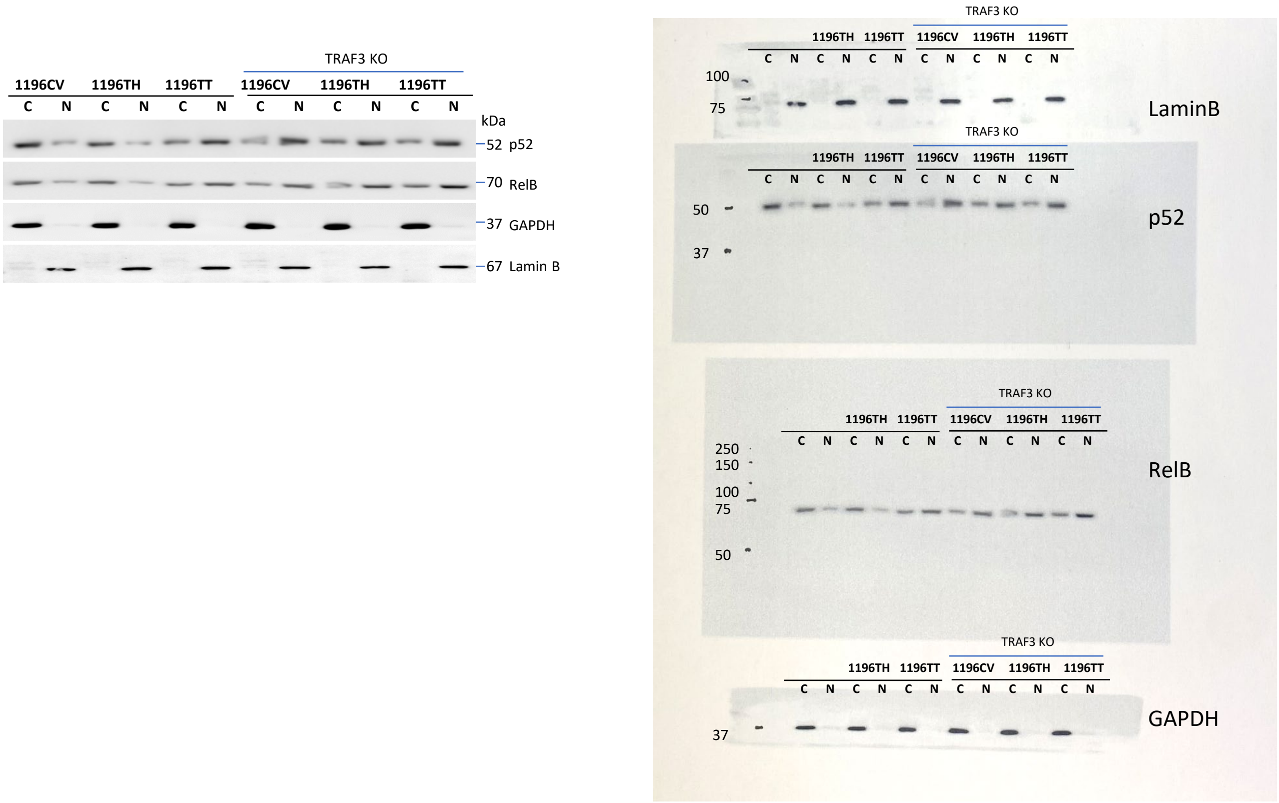
TRAF3 KO
1196CV
1196TH
1196TT
1196CV
1196TH
1196TT
C N C N C N C N C N C N
kDa
52 p52
70 RelB
37 GAPDH
67 Lamin B
TRAF3 KO
1196TH 1196TT 1196CV 1196TH 1196TT
C N C N C N C N C N C N
100
75
LaminB
TRAF3 KO
1196TH 1196TT 1196CV 1196TH 1196TT
C N C N C N C N C N C N
50
37
p52
TRAF3 KO
1196TH 1196TT 1196CV 1196TH 1196TT
C N C N C N C N C N C N
250
150
100
75
50
RelB
TRAF3 KO
1196TH 1196TT 1196CV 1196TH 1196TT
C N C N C N C N C N C N
37
GAPDH

3b

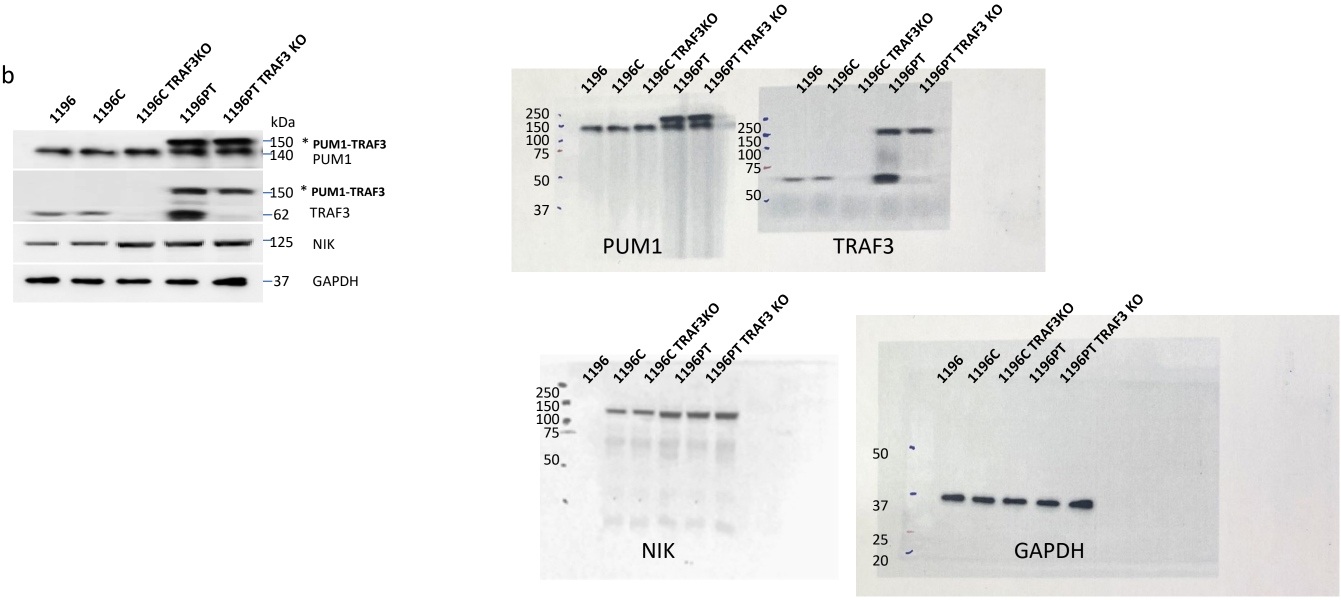
1196
1196C
1196C TRAF3KO
1196PT
1196PT TRAF3 KO
kDa
150 * PUM1-TRAF3
140 PUM1
150 * PUM1-TRAF3
62 TRAF3
125 NIK
37 GAPDH
250
150
100
75
50
37
PUM1
250
150
100
75
50
TRAF3
250
150
100
75
50
NIK
50
37
25
20
GAPDH

3c

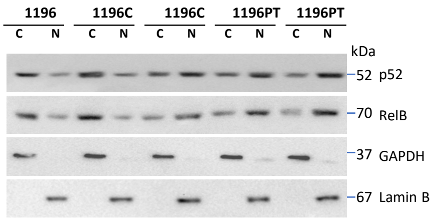

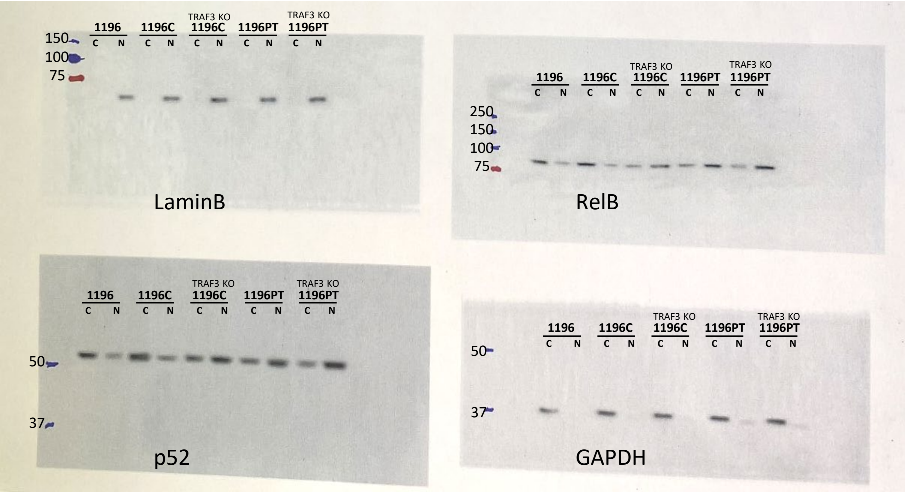

4a

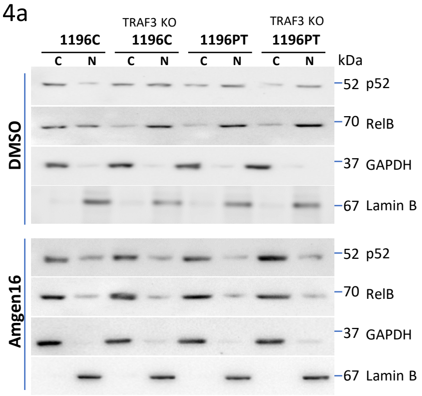

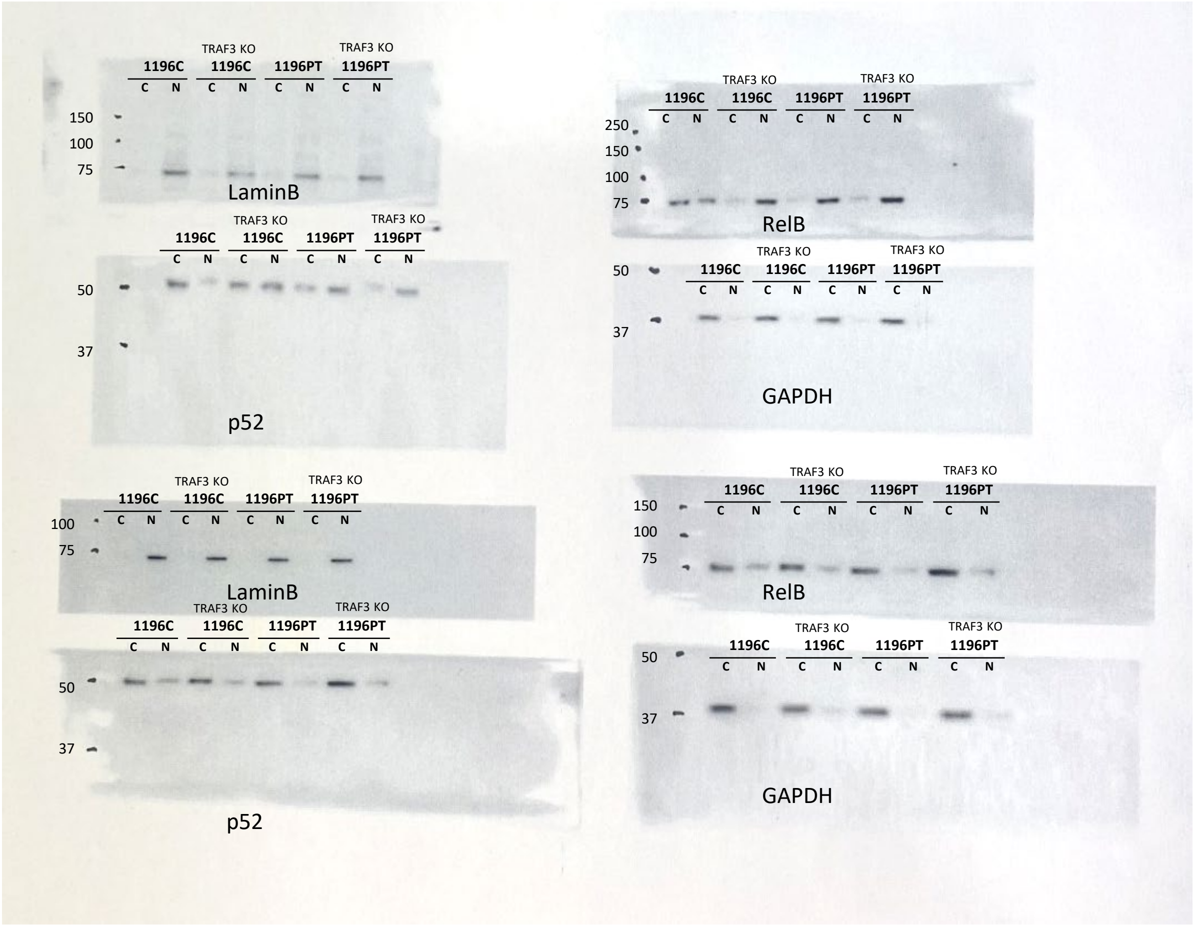

Supplementary Fig. 11. Western blots

Supplementary Table 1. Characteristics of the patients in the discovery group

Patient	Age	Sex	Primary site	Operation	Pathology	Differentiation	Stage	RM	Adjuvant therapy	DFS, mo	OS,mo
**B01**	66	Female	intrahepatic	Lobectomy of liver	Cholangiocarcinoma	Moderate	T2N1M0	R0	Yes	13.7	25.6
**B02**	72	Female	intrahepatic	Lobectomy of liver	Cholangiocarcinoma	Moderate	T3N1M0	R0	Yes	16.2	23.1
**B03**	77	Female	extrahepatic	PPPD	Cholangiocarcinoma	Moderate	T2N0M0	R0	No	17.8	19.1
**B04**	80	Male	intrahepatic	Lobectomy of liver	Cholangiocarcinoma	Poor	T2N1M0	R0	Yes	8.9	10.8
**B05**	67	Male	extrahepatic	PPPD	Cholangiocarcinoma	Moderate	T3N0M0	R0	Yes	11.2	18.2

Abbreviation: PPPD, Pylorus preserving pancreatoduodenectomy; RM, Resection margin; DFS, Disease free survival; OS, Overall survival; mo, months.

Supplementary Table 2. Primers for RT-PCR, qRT-PCR, ddPCR, and gene cloning

Primers for PCR

Gene	Direction	Sequences
Pum1-Traf3_PT1	Forward	5'- TGT ATG GCT GCC GTG TTA TC -3'
Pum1-Traf3_PT1	Reverse	5'- ATG TCG TGC ACA CTC AGC AT -3'
Pum1-Traf3_PT2	Forward	5'- GCA ATC ACG TGG TTC AGA AA-3'
Pum1-Traf3_PT2	Reverse	5'- TGC TCT TCA TGC TGT CTG CT-3'
GAPDH	Forward	5'- GTC TCC TCT GAC TTC AAC AGC G -3'
GAPDH	Reverse	5'- ACC ACC CTG TTG CTG TAG CCA A -3'

Primers for ddPCR (same sequences used for PCR)

Gene	Direction	Sequences
Pum1-Traf3_PT2	Forward	5'- GCA ATC ACG TGG TTC AGA AA-3'
Pum1-Traf3_PT2	Reverse	5'- TGC TCT TCA TGC TGT CTG CT-3'

Primers for gene cloning

Gene	Direction	Sequences
Pum1-Traf3_PT3	Forward	5'- CCA TGT TGT CGG AGT GAA AG-3'
Pum1-Traf3_PT3	Reverse	5'- AGA CAG ACC GGT TCA AAT CC-3'
Pum1-Traf3_PT4	Forward	5'- ATT AAG CTT GCG GCC ATG AGC GTT GCA TGT GTC TT-3'
Pum1-Traf3_PT4	Reverse	5'- GAT GAA TTC GCG GCC TTG GGA TCG GGC AGA TCC G-3'

Supplementary Table 3. Antibodies for western blot, immunohistochemistry, immunofluorescence, proximity ligation analysis, and FACS

Antibodies	Suppliers	Species	Product Number	Experiments (dilution)
TRAF2	Santa Cruz Biotechnology	Mouse monoclonal	SC-136999	WB (1:1000)
TRAF2	Santa Cruz Biotechnology	Rabbit polyclonal	SC-876	IP (1μg per 100μg)
TRAF3	Santa Cruz Biotechnology	Mouse monoclonal	SC-6933	PLA (1:200)<br>WB (1:1000)
TRAF3	Santa Cruz Biotechnology	Rabbit polyclonal	SC-949	IP (1μg per 100μg)
PUM1	Santa Cruz Biotechnology	Rabbit polyclonal	SC-135049	PLA (1:200)<br>WB (1:1000)<br>IP (1μg per 100μg)
P27/Kip-1(Ab-2)	Merck Millipore	Mouse monoclonal	NA35-100UG	WB (1:1000)
NF-κB2 (p52)	Santa Cruz Biotechnology	Mouse monoclonal	SC-7386	WB (1:1000)<br>IHC, IF, PLA (1:200)
RelB	Santa Cruz Biotechnology	Mouse monoclonal	SC-48366	IHC (1:200)
RelB	Santa Cruz Biotechnology	Rabbit polyclonal	SC-226	WB (1:1000)<br>IF, PLA (1:200)
phopho-IKKαβ	Santa Cruz Biotechnology	Rabbit polyclonal	SC-21661-R	WB (1:1000)
IKKα	Cell Signaling	Mouse monoclonal	11930	WB (1:1000)
NIK	Santa Cruz Biotechnology	Mouse monoclonal	SC-8417	WB (1:1000)<br>IF (1:200)
NIK	Abcam	Rabbit polyclonal	ab155583	WB (1:1000)<br>IHC (1:200)
NIK	Abcam	Rabbit polyclonal	ab80418	IP (1μg per 100μg)
N-Cadherin	Abcam	Mouse monoclonal	ab98952	WB (1:1000)
E-Cadherin	Santa Cruz Biotechnology	Rabbit polyclonal	SC-7870	WB (1:1000)
TWIST	Santa Cruz Biotechnology	Rabbit polyclonal	SC-15393	WB (1:1000)
Snail/Slug	Abcam	Rabbit polyclonal	ab85936	WB (1:1000)
OCT4	Santa Cruz Biotechnology	Rabbit polyclonal	SC-9081	WB (1:1000)
GAPDH	Santa Cruz Biotechnology	Mouse monoclonal	SC-47724	WB (1:1000)
LaminB	Santa Cruz Biotechnology	Goat polyclonal	SC-6216	WB (1:1000)
GFP	Millipore Sigma	Mouse monoclonal	MAB3580	WB (1:1000)
CD133/2-APC	Miltenyi Biotech	Mouse monoclonal	130-113-106	FC (1μg per $1x10^6$ cells)
Isotype Control IgG1-APC	Miltenyi Biotech	Mouse monoclonal	130-113-196	FC (1μg per $1x10^6$ cells)
Normal rabbit IgG	Santa Cruz Biotechnology	Rabbit	SC-2027	IP (1μg per 100μg)<br>PLA (1:200)
Normal mouse IgG	Santa Cruz Biotechnology	Mouse	SC-2025	IP (1μg per 100μg)<br>PLA (1:200)
Anti-rabbit IgG, HRP-linked Antibody	Cell Signaling	Goat anti-rabbit	7074	WB (1:5000)
Donkey anti-mouse IgG-HRP	Santa Cruz Biotechnology	Donkey anti-mouse	SC-2314	WB (1:5000)
Anti-mouse IgG (H+L), F(ab')2 Fragment (Alexa Fluor® 555 Conjugate)	Cell Signaling	Goat anti-mouse	4409	IF (1:200)
Anti-rabbit IgG (H+L), F(ab')2 Fragment (Alexa Fluor® 555 Conjugate)	Cell Signaling	Goat anti-rabbit	4413	IF (1:200)

Abbreviation: WB, western blot; IP, immunoprecipitation; PLA, proximity ligation assay; IHC, immunohistochemistry; IF, immunofluorescence staining; FC, flow cytometry.

Supplementary Table 4. Baseline characteristics of the patients

	Fusion gene (-)<br>n= 50 (90.9%)	Fusion gene (+)<br>N = 5 (9.1%)
Age at diagnosis, Mean (SD)	67.0 (±9.5)	68.7 (±7.2)
Gender (%)		
Female	18 (36%)	5 (100%)
Male	32 (64%)	0 (0%)
Origin of tumor (%)		
Intrahepatic CC	6 (12%)	3 (60%)
Extrahepatic CC	43 (86%)	1 (20%)
GB cancer	1 (2%)	1 (20%)
Stage (%)		
I	14 (28%)	1 (20%)
II	31 (62%)	1 (20%)
III	3 (6%)	2 (40%)
IVa	2 (4%)	1 (20%)
[I + II]	45 (90%)	2 (40%)
[III + IVa]	5 (10%)	3 (60%)
Differentiation (%)		
Well	6 (12%)	1 (20%)
Moderate	31 (62%)	3 (60%)
Poor	11 (22%)	2 (40%)
Undifferentiated	2 (4%)	0 (0%)
CA 19-9, IU/mL, median (range)	997.1 (±2910.2)	311.4 (±865.2)
Resection margin (%)		
R0	11 (22%)	4 (80%)
R1	39 (78%)	1 (20%)
Adjuvant chemotherapy (%)		
Yes	27 (54%)	4 (80%)
No	23 (46%)	1 (20%)
Recurrence after surgery (%)		
Yes	25 (50%)	4 (80%)
No	25 (50%)	1 (20%)
Distant metastasis at recurrence (%)†	22 (88%)	4 (100%)
Liver	14 (56%)	1 (25%)
Lung	5 (20%)	0 (21.1%)
Brain	2 (8%)	2 (50%)
Peritoneum	4 (16%)	3 (75%)
Palliative chemotherapy after recurrence (%)		
Yes	7/11 (63.6%)	15/18 (83.3%)
No	4/11 (36.4%)	3/18 (16.7%)
Survival, months, median (range)		
Disease free survival	16.2 (0.2-122.5)	12.7 (1.4-32.4)
Overall survival	34.9 (1.2-122.7)	29.6 (6.5-45.2)

† The ratio in patients with recurrence and multiple organ metastases was counted separately.

Abbreviation: SD, standard deviation; CC, cholangiocarcinoma.

Supplementary Table 5. Characteristics of the patients with PUM1-TRAF3 FISH positive

Patient	Age	Sex	Primary site	Differentiation	Stage	RM	Adjuvant therapy	Recurrence	DFS, mo	OS, mo
**P1**	65	Female	intrahepatic	Well	T2N0M0	R0	Yes	Yes	12.7	45.2
**P5**	75	Female	intrahepatic	Poor	T2N1M0	R0	Yes	Yes	25.9	29.6
**P11**	70	Female	intrahepatic	Moderate	T1N0M0	R0	No	No	32.4	32.9
**P12**	71	Female	Gallbladder	Moderate	T3N0M0	R0	Yes	Yes	7.2	15.1
**P36**	76	Female	extrahepatic	Moderate	T2N1M0	R1	Yes	Yes	1.4	6.5
